# Engineering *Geobacillus thermoglucosidasius* for direct utilisation of holocellulose from wheat straw

**DOI:** 10.1186/s13068-019-1540-6

**Published:** 2019-08-20

**Authors:** Zeenat Bashir, Lili Sheng, Annamma Anil, Arvind Lali, Nigel P. Minton, Ying Zhang

**Affiliations:** 1 Clostridia Research Group, BBSRC/EPSRC Synthetic Biology Research Centre (SBRC), School of Life Sciences, University of Nottingham, University Park, Nottingham, NG7 2RD UK; 2 DBT-ICT Centre for Energy Biosciences, Institute of Chemical Technology, Nathalal Parikh Marg, Mumbai, 400019 India

**Keywords:** *Geobacillus thermoglucosidasius*, Biomass, Consolidated bioprocessing (CBP), Glycoside hydrolases, Cellulases, Endo/exoglucanases, β-Glucosidase

## Abstract

**Background:**

A consolidated bioprocessing (CBP), where lignocellulose is converted into the desired product(s) in a single fermentative step without the addition of expensive degradative enzymes, represents the ideal solution of renewable routes to chemicals and fuels. Members of the genus *Geobacillus* are able to grow at elevated temperatures and are able to utilise a wide range of oligosaccharides derived from lignocellulose. This makes them ideally suited to the development of CBP.

**Results:**

In this study, we engineered *Geobacillus thermoglucosidasius* NCIMB 11955 to utilise lignocellulosic biomass, in the form of nitric acid/ammonia treated wheat straw to which expensive hydrolytic enzymes had not been added. Two different strains, BZ9 and BZ10, were generated by integrating the *cglT* (β-1,4-glucosidase) gene from *Thermoanaerobacter brockii* into the genome, and localising genes encoding different cellulolytic enzymes on autonomous plasmids. The plasmid of strain BZ10 carried a synthetic cellulosomal operon comprising the *celA* (Endoglucanase A) gene from *Clostridium thermocellum* and *cel6B* (Exoglucanase) from *Thermobifida fusca*; whereas, strain BZ9 contained a plasmid encoding the *celA* (multidomain cellulase) gene from *Caldicellulosiruptor bescii*. All of the genes were successfully expressed, and their encoded products secreted in a functionally active form, as evidenced by their detection in culture supernatants by Western blotting and enzymatic assay. In the case of the *C. bescii* CelA enzyme, this is one of the first times that the heterologous production of this multi-functional enzyme has been achieved in a heterologous host. Both strains (BZ9 and BZ10) exhibited improved growth on pre-treated wheat straw, achieving a higher final OD600 and producing greater numbers of viable cells. To demonstrate that cellulosic ethanol can be produced directly from lignocellulosic biomass by a single organism, we established our consortium of hydrolytic enzymes in a previously engineered ethanologenic *G. thermoglucosidasius* strain, LS242. We observed approximately twofold and 1.6-fold increase in ethanol production in the recombinant *G. thermoglucosidasius* equivalent to BZ9 and BZ10, respectively, compared to *G. thermoglucosidasius* LS242 strain at 24 h of growth.

**Conclusion:**

We engineered *G. thermoglucosidasius* to utilise a real-world lignocellulosic biomass substrate and demonstrated that cellulosic ethanol can be produced directly from lignocellulosic biomass in one step. Direct conversion of biomass into desired products represents a new paradigm for CBP, offering the potential for carbon neutral, cost-effective production of sustainable chemicals and fuels.

**Electronic supplementary material:**

The online version of this article (10.1186/s13068-019-1540-6) contains supplementary material, which is available to authorized users.

## Background

The sustainable production of chemicals and fuels from lignocellulosic biomass using microbial fermentation requires its deconstruction into simple sugars. This conversion is dependent on an initial pre-treatment step followed by the addition of hydrolytic enzymes. Pre-treatment can be accomplished using a variety of methods such as biological, chemical, mechanical and thermal processes, either alone or in combination [1, 2]. The subsequent complete hydrolysis of the cellulose in pre-treated biomass into glucose, however, requires the synergetic action of at least three types of glycoside hydrolases (GHs) that belong to different sequence-based families, as classified in the Carbohydrate-Active Enzyme database (CAZy) [3]. Endoglucanases hydrolyse internal bonds in the cellulose, generating short polymers which provide the substrate for cellobiohydrolases (or exoglucanases). These act in a unidirectional manner, either from non-reducing or reducing ends of cellulose polysaccharide chains, liberating cellobiose as the major product. Lastly, β-glucosidases convert cellobiose into glucose, relieving the system from end product inhibition [4]. The relative expense of pre-treatment steps, coupled with high cost of hydrolytic enzymes, has made the development of cost-effective strategies to produce chemicals and fuels from biomass very challenging.

One solution to improve the economics is consolidated bioprocessing (CBP), in which lignocellulose is converted into products in one step without added enzymes. In essence, CBP represents the simultaneous enzyme-mediated saccharification and fermentation of biomass into desired products by a single microorganism. Although, no natural microorganism possesses all of the desired features of CBP, certain bacteria and fungi possess at least some of the properties required [5–7]. Of particular interest are thermophilic microorganisms because of the following features: (i) they produce thermostable enzymes that can survive under the required harsh, bioprocessing conditions [8]; (ii) they grow at high temperatures that promotes higher rates of feedstock conversion [9]; (iii) the risk of contamination is eliminated at higher processing temperatures, which also reduce viscosity of substrate and product streams [10, 11], and; (iv) high temperature assists product removal and recovery, potentially lowering separation cost [12]. Among the thermophiles, the anaerobic, cellulolytic organism *Clostridium thermocellum* is one of the most extensively researched CBP organisms and effectively degrades cellulose using a multiprotein complex called the cellulosome. In its wild-type form, *C. thermocellum* is unable to catabolize pentose sugars resulting from the hydrolysis of hemicellulose, thereby reducing the overall biomass conversion. Its low ethanol tolerance also limits the levels of this biofuel that can be produced [6, 13–15]. Other hemicellulose-utilising thermophilic microorganisms belonging to the genus, *Thermoanaerobacterium* and *Thermoanaerobacter,* have been developed as a CBP organism but are similarly limited in ethanol yields [16–18]. Recently, an extremely cellulolytic, thermophilic organism, *Caldicellulosiruptor bescii*, was engineered to produce an ethanol [19]. Although an important step towards establishing *C. bescii* as a platform for CBP, the high (78 °C) optimum growth temperature of *C. bescii* present a number of challenges to further metabolic engineering the organism for extend product range. These include the existence of a very limited number of antibiotics that are stable above 50 °C, and the lack of available genetic tools [20, 21].

Members of the genus *Geobacillus* are moderate thermophiles (optimum growth temperature is 55–60 °C) that have also been promoted as potential platforms for CBP. They can grow to a high cell density and can utilise a wide range of polymeric or short oligomeric carbohydrates for growth [22]. Importantly, in recent years, genetic tools have been developed that have enabled them to be engineered to improve the production of natural products, such as ethanol [23–26], as well as non-native products, such as isobutanol [27]. However, while most of the *Geobacillus* spp. can effectively degrade hemicellulose, they are unable to break down crystalline or the amorphous cellulose [28], despite their possession of numerous endoglucanases and endoxylanases [29–31]. This has led to the introduction of heterologous cellulase genes [32, 33] but the encoded GHs were only shown to be active against synthetic substrates such as carboxy-methyl cellulose (CMC), phosphoric acid-swollen cellulose (PASC) and xylan. The introduction of GHs that are better able to degrade natural cellulosic substrate is essential if *Geobacillus* is to form the basis of CBP.

**Fig. 1:**
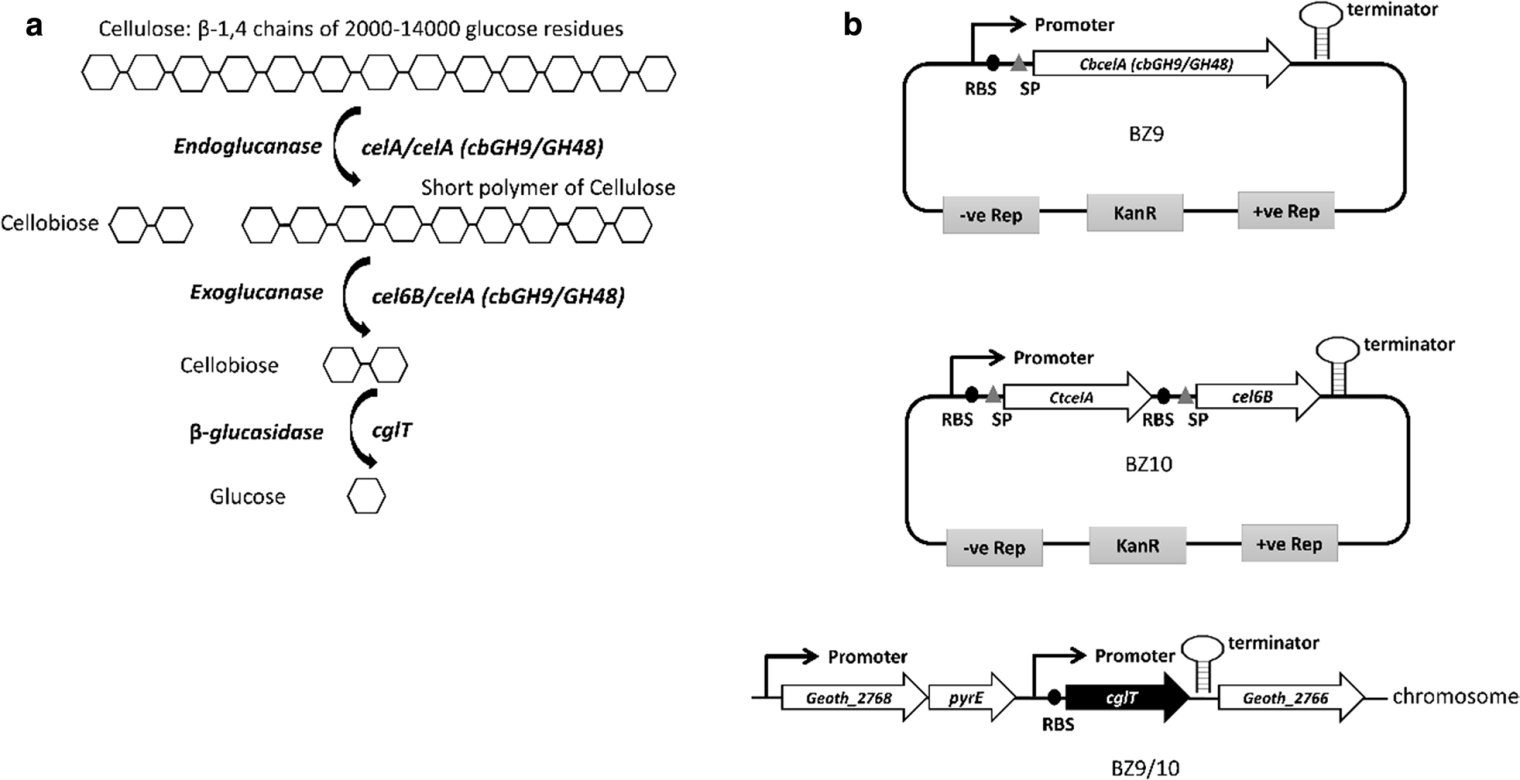
Engineering *G. thermoglucosidasius* NCIMB 11955 for utilisation of lignocellulosic biomass. **a** The endoglucanase (CtCelA/CbCelA) acts on the low-crystallinity part of the cellulose fibre and create free chain-ends. The exoglucanases (Cel6B/CbCelA) then degrade the sugar chain by removing cellobiose units (dimers of glucose) from the free chain-ends. The released cellobiose units are finally hydrolyzed by β-glucosidases (CglT), releasing glucose. **b** The schematic illustrations of two strains, BZ9 and BZ10 containing chromosomally integrated *cglT* (β-1,4-glucosidase) gene from *Thermoanaerobacter brockii*. Strain BZ9 has a synthetic cellulosomal operon comprising Ct*celA* (Endoglucanase A) gene from *Clostridium thermocellum*, Ribosome binding site (RBS), Signal peptide (SP) and *cel6B* (Exoglucanase) from *Thermobifida fusca* heterologously expressed on a replicating plasmid having two origin of replications for Gram-negative (−ve rep) and Gram-positive (+ve Rep) bacteria under the constitutive strong promoter P_*ldh*_. Strain BZ10 contains Cb*celA* (cellulase comprises a glycoside hydrolase family 9 and a family 48 catalytic domain) gene from *Caldicellulosiruptor bescii* on a replicating plasmid

The standard commercial cellulosic enzyme cocktails for biomass deconstruction contains cellobiohydrolase/exoglucanases I (CBH I), cellobiohydrolase/exoglucanases II (CBH II) and β-glucosidase that act synergistically to release sugars for microbial conversion to products [34]. We, thus, anticipated that *G. thermoglucosidasius* recombinant strains endowed with the ability to produce an enzyme cocktail that could mimic the activity of the above would be potentially able to breakdown hemicellulose/cellulose and support growth of organism. Accordingly, in the present study, *G. thermoglucosidasius* NCIMB 11955 was endowed with the necessary thermostable exoglucanase, endoglucanase and β-glucosidase activities to allow its growth on pre-treated wheat straw that had received no enzymatic treatment (Fig. 1). Having established these defined modifications supported growth on a natural cellulosic substrate, they were introduced into a strain previously engineered for ethanol production. This enabled the one-step production of ethanol from the lignocellulose using single recombinant *G. thermoglucosidasius* strains, representing a significant step towards the generation of a platform for CBP.

## Results

### Heterologous expression of extracellular GHs

The codon-optimised Ct*celA, cel6B* and Cb*celA* genes from *C. thermocellum, T. fusca* YX and *C. bescii*, respectively, were cloned into the *G. thermoglucosidasius* expression vector pMTLgSlimS under the transcriptional control of constitutive P_*ldh*_ promoter, and the resulting plasmids, pMTLgSlimS–Ct*celA*, pMTLgSlimS–*cel6B* and pMTLgSlimS–Cb*celA* were transformed into *G. thermoglucosidasius* NCIMB 11955. The extracellular CtCelA, Cel6B and CbCelA were produced along with their native secretion signals containing a FLAG-epitope at the carboxy-terminus.

**Fig. 2:**
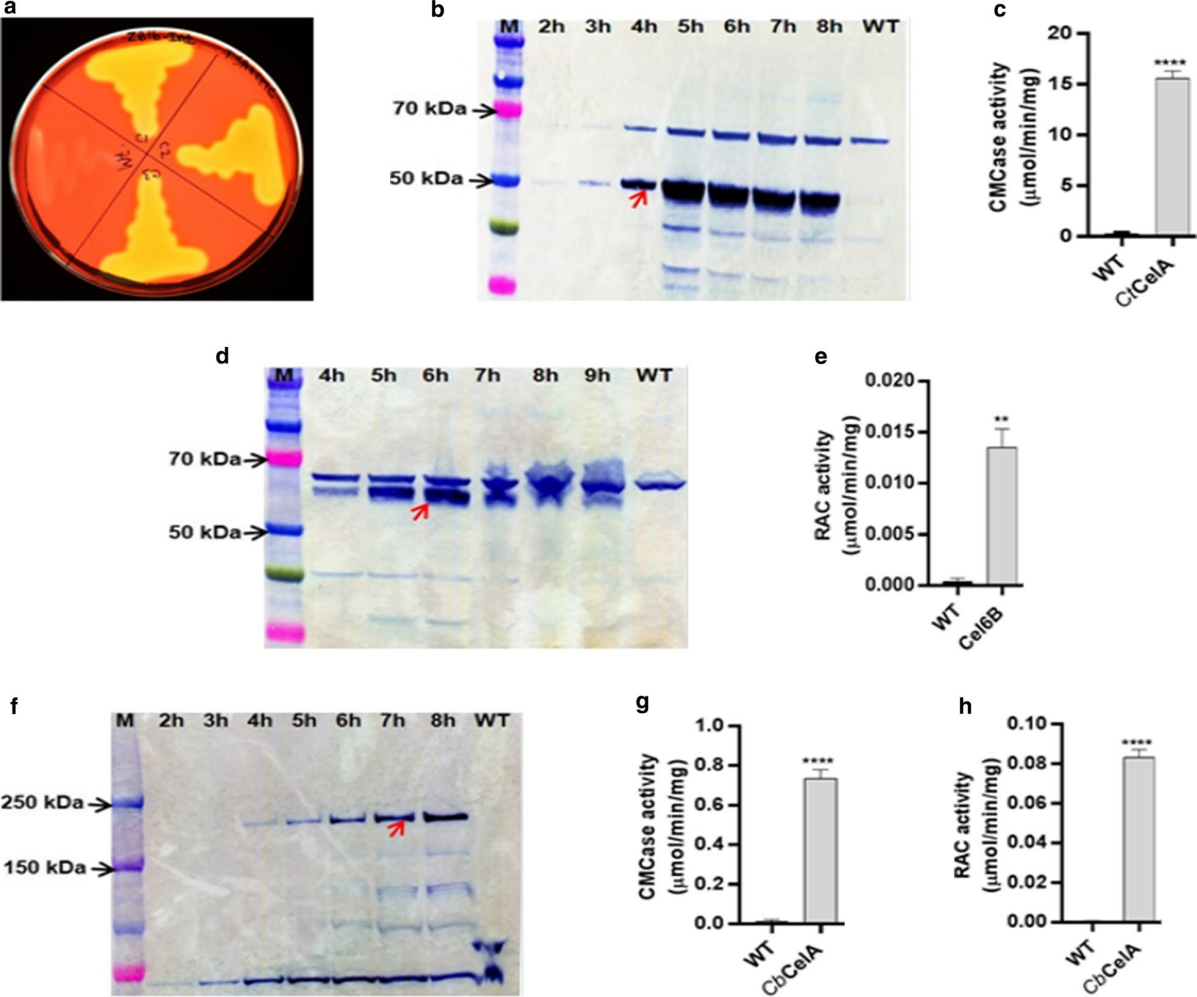
Expression of heterologous GHs by recombinant *G. thermoglucosidasius* strains. The concentrated supernatants from wild type as well as recombinant *G. thermoglucosidasius* strains expressing GHs either CtCelA or Cel6B or CbCelA were separated in SDS-PAGE and extracellular heterologous GHs at the indicated time points (2–9 h) were detected by Western blotting using ANTI-FLAG M2 monoclonal antibody-horseradish peroxidise conjugate. WT represents concentrated supernatant from wild-type strain of *G. thermoglucosidasius* at 8 h; M represents the pre-stained protein ladder (10–250 kDa). Expected molecular mass of the proteins are indicated by red arrows. **a** Congo red-stained TSA-CMC agar plate streaked with recombinant *G. thermoglucosidasius* expressing CtCelA enzyme (zone C1, C2, C3) and wild-type strain (zone WT). **b** Western blot showing the 100-fold concentrated supernatants from recombinant strain expressing the 50 kDa CtCelA. **c** CMCase specific activity of extracellular fraction of recombinant *G. thermoglucosidasius* expressing CtCelA and wild-type *G. thermoglucosidasius* strains (WT). **d** Western blot showing the 100-fold concentrated supernatants from recombinant *G. thermoglucosidasius* expressing the 60 kDa Cel6B. **e** RACase specific activity of extracellular fraction of recombinant *G. thermoglucosidasius* expressing Cel6B and *G. thermoglucosidasius* wild-type (WT) strains. **f** Western blot showing the 200-fold concentrated supernatants of recombinant *G. thermoglucosidasius* expressing the 193 kDa CbCelA. **g** CMCase and **h** RACase specific activity of extracellular fraction of recombinant *G. thermoglucosidasius* CbCelA and wild-type *G. thermoglucosidasius* strains (WT). *P* values were calculated by student’s *t* test and results are shown as mean ± SEM of three biological replicates

To examine CtCelA production and its endoglucanase activity, single recombinant *G. thermoglucosidasius* colonies, as well as the wild-type strain, were tested for CMC hydrolysis on appropriate agar media. The clear zone around the recombinant strains demonstrated that CMC hydrolysis as a result of the secretion of endoglucanase was occurring. In contrast, parent strain lacked any activity by the same measure (Fig. 2a). To analyze the expression of CtCelA, Cel6B and CbCelA in recombinant *G. thermoglucosidasius* strains, anti-FLAG M2-peroxidase (HRP) antibody antibody was used in Western blots of concentrated culture supernatants collected every hour until the stationary phase of growth (OD600 > 7). The heterologous CtCelA protein at an estimated molecular weight of 50 kDa was clearly visible in the 100-fold concentrated supernatant after 3 h, reaching a maximum in 5 h. No such band was observed in the supernatant derived from the wild-type strain (Fig. 2b). An electrophoretic band that bound to the anti-FLAG antibodies equivalent to a protein of 65 kDa was evident in the supernatants derived from both the recombinant and wild-type strains suggesting that strain NCIMB 11955 contains a polypeptide that also binds to the anti-FLAG-antibody used. A few weak bands of a size smaller than 50 kDa were also apparent in the recombinant strain but absent in wild type. It is possible that this was due to degradation of CtCelA protein in extracellular medium.

Cell growth is often inhibited because of the metabolic burden imposed by the expression of heterologous protein genes [35]. However, in the present study, the growth profile of the recombinant *G. thermoglucosidasius* strains harbouring plasmid pMTLgSlimS–Ct*celA* was similar to that of wild type (Additional file 1: Figure S1). CMCase/endoglucanase activity of the recombinant *G. thermoglucosidasius* strains expressing CtCelA was found to be 15.6 ± 1 μmol min^−1^ mg^−1^ of protein confirming that the protein is functional (Fig. 2c).

The presence of recombinant Cel6B protein of an estimated molecular weight of 60 kDa was confirmed from the Western blotting of 100-fold concentrated supernatant derived from recombinant *G. thermoglucosidasius* harbouring plasmid pMTLgSlimS-*cel6B* (Fig. 2d). This observation confirmed the successful expression and secretion of Cel6B. To assess the exoglucanase activity of the heterologously produced Cel6B, enzyme assays using RAC (Regenerated Phosphoric Acid-Swollen Cellulose) as substrate were performed. From this, specific activity of Cel6B was estimated to be 0.0135 ± 0.003 μmol min^−1^ mg^−1^ of protein. No measurable activity was detected in supernatants derived from the wild type (Fig. 2e).

To confirm the full-length expression of CbCelA protein, 200-fold concentrated supernatant was analysed. Using Western Blotting and anti-FLAG antibodies, we detected a protein with an estimated molecular mass of 193 kDa in the supernatant of the recombinant *Geobacillus* culture after 4 h of growth. This corresponded to the estimated molecular weight of CbCelA and confirmed the successful production of this glycosyl hydrolase in a soluble form (Fig. 2f). Although the calculated molecular mass of CbCelA protein is 193 kDa, other studies have found that the native protein from *C. bescii* migrates on SDS-PAGE at an apparent molecular weight of 230 kDa as a consequence of the glycosylation of native enzyme [36, 37]. However, our results showed a band size of 193 kDa indicating the absence of glycosylation in the heterologously expressed CbCelA protein. Endoglucanase and exoglucanase functionality assays of the recombinant enzyme produced in *Geobacillus* indicated that specific activity of CbCelA against CMC and RAC was 0.735 ± 0.07 and 0.0831 ± 0.006 μmol min^−1^ mg^−1^ of protein, respectively, suggesting that CbCelA protein is functional (Fig. 2g, h).

In total, all the three enzymes (CtCelA, Cel6B and CbCelA) were, therefore, produced successfully in their soluble form in the *G. thermoglucosidasius* chassis, with measurable enzyme activity.

### Genome integration of the *cglT* gene to improve cellobiose utilisation

The *G. thermoglucosidasius* NCIMB 11955 genome sequence contains an operon encoding a predicted cellobiose specific phosphotransferase system (PTS) followed by a gene annotated as β-1,4-glucosidase indicating that the organism can grow on cellobiose. However, the β-glucosidase operon is tightly regulated by *G. thermoglucosidasius* NCIMB 11955, which leads to low production of enzymes and inefficient degradation of cellobiose. Accumulation of this disaccharide results in feedback inhibition of many exo- and endo-glucanases [38, 39]. To ensure the maximum rate of cellobiose conversion to glucose, an additional β-glucosidase gene (*cglT* from *T. brockii*) was integrated into the chromosome of *G. thermoglucosidasius* via ACE (Allele-Coupled Exchange) [26]. Integration of the *cglT* at the *pyrE* locus was achieved by transforming plasmid pMTLgSlimS–LS3–*cglT* into electro-competent cells of a ∆*pyrE* deletion mutant of *G. thermoglucosidasius* NCIMB 11955 and subsequentially selecting for uracil prototrophs [26]. Prototrophic colonies were further screened for the presence of the correctly integrated P_*ldh*_–*cglT* cassette by colony-PCR analysis using oligonucleotide primers pyrE_C1_F and pyrE_C2_R which anneal to the genomic sequences up- and downstream of the *pyrE* locus. Correct integration of the P_*ldh*_–*cglT* cassette should result in the amplification of a DNA fragment of 3.8 kb in size; while in the case of the parental strain, the same primers generate a 2.0-kb fragment. All clones tested were found to generate the expected 3.8-kb fragment (Additional file 1: Figure S2) confirming the integration of *cglT* gene into the chromosome. The recombinant, integrant strain obtained was designated *G*. *thermoglucosidasius* ZB3bInt.

**Fig. 3:**
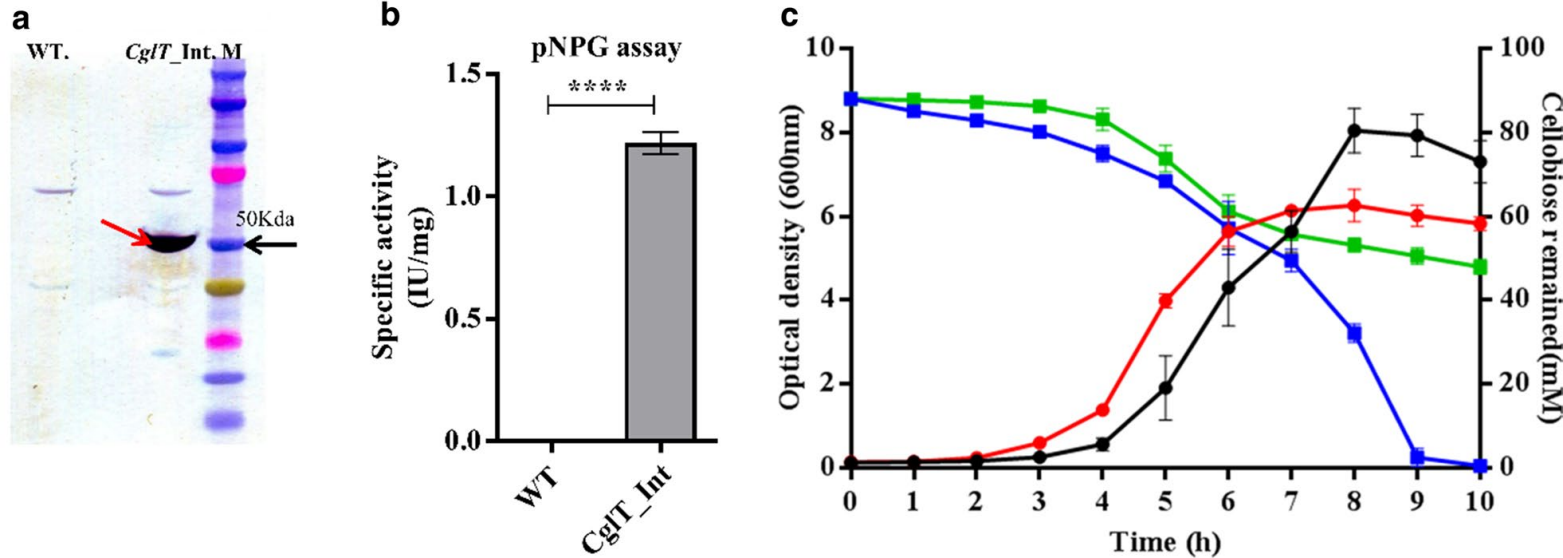
Intracellular expression of CglT enhanced cellobiose utilisation. **a** Western blot showing FLAG-tagged CglT using ANTI-FLAG M2 monoclonal antibody-horseradish peroxidise conjugate. Lane Wt. and CglT represents soluble fraction of cell lysate from wild-type *G. thermoglucosidasius* and the recombinant *G. thermoglucosidasius* ZB3bInt strain, respectively. Lane M is pre-stained protein ladder (10–250 kDa); Expected molecular mass of 50 kDa for CglT is indicated by red arrow. **b** pNPGase specific activity of recombinant *G. thermoglucosidasius* ZB3bInt strains (ZB3bInt) and wild-type *G. thermoglucosidasius* strains (WT). *P* values were calculated by student’s t test and results are shown as mean ± SEM of three biological replicates. **c** Cellobiose consumption and growth profiles by recombinant *G. thermoglucosidasius* ZB3bInt (ZB3bInt) and wild-type *G. thermoglucosidasius* strains (WT) when grown on 3.0% cellobiose as the carbon source at 55 °C. Black circles, *G. thermoglucosidasius*_ZB3bInt (OD600 nm); Red circles, wild-type (OD600 nm); blue square, *G. thermoglucosidasius*_Zb3bInt (remaining cellobiose); green squares, wild-type (remaining cellobiose). Results are shown as mean ± SEM of three biological replicates

To investigate the expression and functionality of intracellular CglT in *G. thermoglucosidasius* ZB3bInt, 80 µg of total protein from a cell-free extract (CFE) was resolved on SDS-PAGE followed by anti-FLAG-tag mediated detection of protein by Western blotting. A distinct protein band estimated to be 50 kDa in size, corresponding to the predicted molecular mass of CglT protein, was observed in supernatants derived from the integrant strain (Fig. 3a). An equivalently size protein was absent in supernatants derived from the wild-type strain. Further, a CFE of *G. thermoglucosidasius* ZB3bInt expressing *cglT* gene showed β-glucosidase activity of 1.21 ± 0.07 μmol min^−1^ mg^−1^ of protein against the colorimetric substrate pNPG (4-nitrophenyl β-d-glucopyranoside) at 60 °C (Fig. 3b).

To determine if the integration of *cglT* gene can enhance cellobiose utilisation, the recombinant *G. thermoglucosidasius* ZB3bInt and wild-type strains were grown at 55 °C in ASYE-modified medium supplemented with 30 g/l of cellobiose (87.7 mM) as the sole carbon source. Cellobiose consumption and growth profiles showed that recombinant *G. thermoglucosidasius* ZB3bInt strains utilised 100% of cellobiose available compared to the wild-type strain which utilised only 45 ± 0.02% of total cellobiose after 10 h of growth (Fig. 3c). Although the recombinant ZB3bInt strain exhibited a slight lag in growth compared to wild type in the early exponential phase of growth, after 7 h, its cell density increased significantly and had reached a much higher OD600 at the stationary phase of growth than the wild type (Fig. 3c). These results suggest that the addition of the heterologous *cglT* gene significantly enhanced the ability of *G. thermoglucosidasius* to utilise cellobiose, justifying the use of ZB3bInt as the background strain for the introduction of heterologous exo- and endo-glucanases.

### Assembled GHs enable *G. thermoglucosidasius* to grow on pre-treated wheat straw

**Fig. 4:**
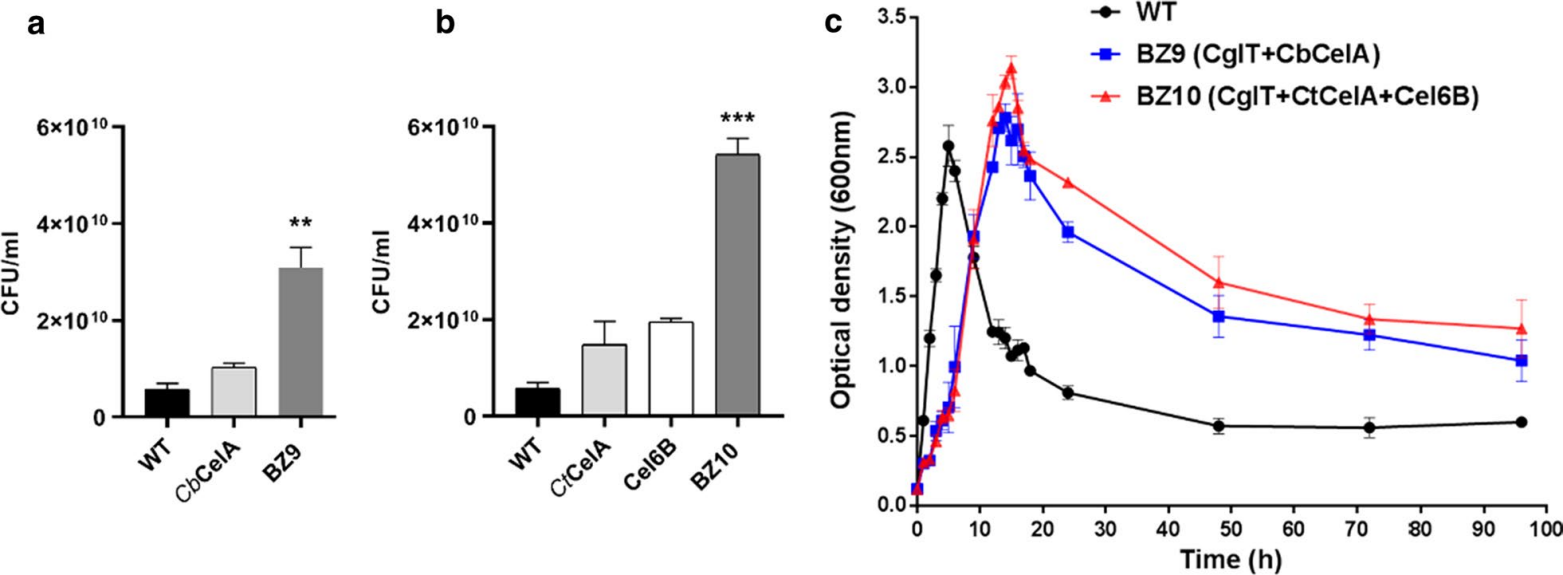
Growth of engineered strains on pre-treated wheat straw. Colony forming units (CFU) were measured after growing recombinant *G. thermoglucosidasius* strains on pre-treated wheat straw for 12 h. **a** CFU comparison of recombinant *G. thermoglucosidasius* expressing only CbCelA (strain ZB6d) with *G. thermoglucosidasius* BZ9 strain expressing CglT and CbCelA. **b** CFU comparison of recombinant *G. thermoglucosidasius* BZ10 strain expressing CglT, Cel6B and CtCelA with *G. thermoglucosidasius* expressing only either CtCelA or Cel6B. P** ≤* 0.01*, P*** ≤ 0.0001 were calculated by one-way ANOVA followed by Sidak’s multiple comparisons test. **c** Growth curve of recombinant *G. thermoglucosidasius* BZ9 and BZ10 strains on 1% pre-treated wheat straw as a sole carbon source, with wild-type *G. thermoglucosidasius* served as a control. Results are shown as mean ± SEM of three biological replicates

To develop *G. thermoglucosidasius* chassis expressing all the enzymes encoding endoglucanase, exoglucanase and β-glucosidase for complete degradation of cellulose, pMTLgSlimS–Cb*celA* and pMTLgSlimS–Ct*celA*–*cel6B* plasmids were individually transformed into electro-competent cells of *G. thermoglucosidasius* ZB3bInt (expressing the integrated *cglT*), thereby generating recombinant *G. thermoglucosidasius* strain BZ9 and BZ10, respectively. The combined effect of GHs for the degradation of pre-treated wheat straw was investigated by analysing the growth of recombinant *G. thermoglucosidasius* BZ9 and BZ10 with the recombinant strains expressing only either CtCelA or Cel6B or CbCelA, along with wild type as a control. Pre-treated wheat straw was prepared in a two-step process using nitric acid followed by ammonia at the DBT-ICT 2G (Department of Biotechnology—Institute of Chemical Technology Second Generation) Ethanol Demonstration Facility and was kindly gifted by ICT, Mumbai for the study (Additional file 1: Figure S3). It is composed of 85.15% cellulose and 4.35% xylose of the total carbohydrates, as reported in the patent [40]. Cultures of the two recombinant strains BZ9 and BZ10, alongside the wild-type and ZB3bInt controls, prepared in ASYE-modified medium supplemented with 10 g/l pre-treated wheat straw, were grown at 55 °C for approximately 100 h. No growth difference was observed between wild-type and ZB3bInt strains; hence, the wild-type data are included in Fig. 4. Estimates of the number of viable cells present were made throughout growth by counting the number of colony forming units (CFU) obtained when serially dilutions were plated on TSA agar plates. The CFU obtained after 12 h of growth from strain BZ9 was twofold higher compared to strain expressing only CbCelA (Fig. 4a); while, a threefold difference was seen with BZ10 compared to the strains expressing either only CtCelA (strain ZB1b) or Cel6B (strain ZB9a) (Fig. 4b). These results strongly suggest that the combined action of GHs; CtCelA, Cel6B, CglT, and CbCelA has resulted in the production of sufficient simple sugars from pre-treated wheat straw to support improved cell growth and biomass accumulation in recombinant strains BZ9 and BZ10.

Overall, the recombinant BZ9 and BZ10 strains exhibited higher growth than the wild type which was sustained during the whole 96-h period monitored. Although during the first 6 h of growth phase, the rate of growth of the wild type appeared to be greater than BZ9 and BZ10, the wild-type cell density decreased drastically after 8 h, suggesting that the initial growth was supported by the small amount of xylose presented in pre-treated wheat straw. Once this had been consumed, wild-type *G. thermoglucosidasius* could not sustain growth further, as evidenced by the rapid decline in OD600. While the recombinant *G. thermoglucosidasius* BZ9 and BZ10 strains showed a longer lag phase up until the 6-h time point, they went on to achieve a much higher OD600, peaking after the 16-h time point after which there was a gradual decline (Fig. 4c).

To confirm that the recombinant BZ9 and BZ10 strains degrade pre-treated wheat straw by producing GHs, cultures were serially diluted at 24, 48, 72 and 96 h of growth and then plated on TSA-CMC plates. We observed a greater number of colonies, as well as clear zones, on Congo red-stained plates up to the 96-h timepoint in the samples derived from the recombinant BZ9 and BZ10 cultures compared to the wild-type control, where there were less colonies and no indication of CMC degradation. These data confirmed that the recombinant cultures were producing active endoglucanases (CtCelA/CbCelA) (Additional file 1: Figure S4). In addition, we found measurable cellobiohydrolase activity against chromogenic substrate pNPC only in the recombinant *G. thermoglucosidasius* BZ9 and BZ10 strains, confirming the integrity of Cel6B and CbCelA. The specific activity at 24, 48, 72 and 96 h of growth was 0.0279 ± 0.002, 0.0255 ± 0.003, 0.020 ± 0.001 and 0.0187 ± 0.002 μmol min^−1^ mg^−1^ of protein in *G. thermoglucosidasius* BZ9; in *G. thermoglucosidasius* BZ10, the activities were 0.0308 ± 0.01, 0.0226 ± 0.002, 0.0216 ± 0.002, 0.186 ± 0.002 μmol min^−1^ mg^−1^ of protein, respectively (Additional file 1: Figure S5a, b). Overall, these data demonstrated that the two engineered *G. thermoglucosidasius* strains, BZ9 and BZ10, were capable of degrading pre-treated wheat straw as the sole carbon source without the addition of any hydrolytic enzymes.

### Engineering the bioethanol producing *G. thermoglucosidasius* LS242 for biomass utilisation

To demonstrate that cellulosic ethanol can be produced directly from lignocellulosic biomass by a single organism, we next established our consortium of hydrolytic enzymes in a previously engineered ethanologenic *G. thermoglucosidasius* strain, LS242 (∆*ldh*, *pdh*^up^, ∆*pfl*) [26] and tested the ability of the resultant strain to utilise pre-treated wheat straw. To achieve this, *cglT* was integrated into the *pyrE* locus of the *G. thermoglucosidasius* LS242 genome using ACE. This was accomplished as before, through the electrotransformation of LS242 with the plasmid pMTLgSlimS–LS3–*cglT* into *G. thermoglucosidasius* LS004, a ∆*pyrE* deletion mutant of LS242, and selecting for uracil prototrophs. Prototrophic colonies were then screened by colony-PCR analysis using primers pyrE_C1_F and pyrE_C2_R. Correct integration of the P_*ldh*_–*cglT* cassette resulted in the amplification of an expected 3.8-kb product size. Sanger sequencing further confirmed the presence of the predicted nucleotide sequence and the strain was designated *G*. *thermoglucosidasius* BZ242. Subsequently, to construct ethanologenic as well as cellulolytic strains, pMTLgSlimS–Cb*celA* and pMTLgSlimS–Ct*celA*–*cel6B* plasmids were individually transformed into electro-competent cells of *G. thermoglucosidasius* BZ242, thereby generating recombinant *G. thermoglucosidasius* strains BZ243 and BZ244, respectively.

**Fig. 5:**
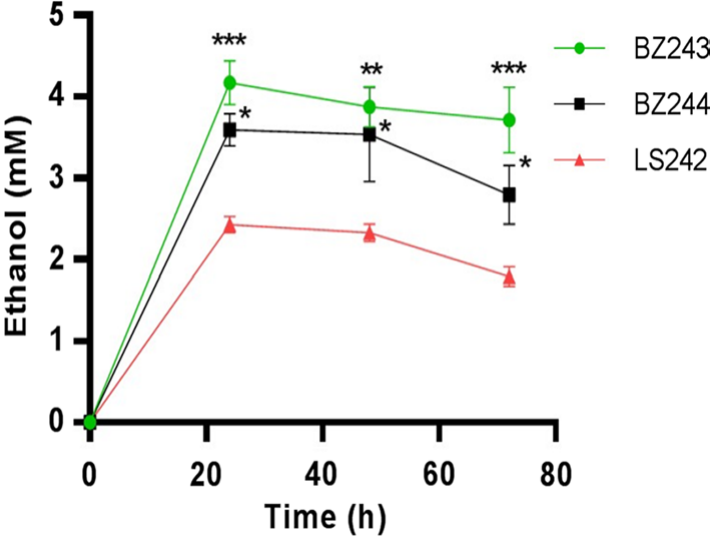
Time course profile for production of ethanol from pre-treated wheat straw by recombinant *G. thermoglucosidasius* BZ243, BZ244 and LS242 strains. P**  ≤ 0.01, P**  ≤ 0.001, P***  ≤ 0.0001 were calculated by 2-way ANOVA followed by Tukey’s multiple comparisons test. Results are shown as mean ± SEM of three biological replicates

To investigate the production of ethanol from pre-treated wheat straw using the recombinant *G. thermoglucosidasius* strains BZ243 and BZ244, together with the engineered ethanologenic *G. thermoglucosidasius* strain LS242 (∆*ldh*, *pdh*^up^, ∆*pfl*) as a control, microaerobic fermentations were carried out as previously described [26]. Culture supernatants from these strains were used for measurement of ethanol production by HPLC at growth time points of 24, 48 and 72 h. We observed approximately twofold (4.2-mM ethanol) and 1.6-fold (3.7-mM ethanol) increase in ethanol production in the recombinant *G. thermoglucosidasius* BZ243 and BZ244 strains, respectively, compared to *G. thermoglucosidasius* LS242 strain (2-mM ethanol) at 24 h of growth. *G. thermoglucosidasius* LS242 strain produced 2-mM ethanol from wheat straw, most likely as a consequence of consumption of xylose present in pre-treated wheat straw. The levels of ethanol in these cultures remained constant up to 48 h and then gradually declined due to the volatile nature of ethanol at temperatures of 55 °C (Fig. 5).

## Discussion

The goal of the study described here was to establish the principle of cellulolytic CBP chassis based on the thermophile *G. thermoglucosidasius*. To that end, we first equipped the otherwise non-cellulolytic, *G. thermoglucosidasius* wild-type strain with the ability to produce heterologous endoglucanase, exoglucanase and β-glucosidase and demonstrated that the engineered recombinant strains were capable of acquiring carbon and energy from pre-treated wheat straw for growth and accumulation of biomass. We then applied this strategy to an engineered ethanologenic *G. thermoglucosidasius* strain, allowing ethanol to be produced directly from lignocellulosic biomass in one step in a single organism.

Based on reported thermophilic activities and properties, the following enzymes were selected to create a cellulolytic *G. thermoglucosidasius*: *C. thermocellum* CtCelA (endoglucanase); *T. fusca* Cel6B (exoglucanase); *C. bescii* CbCelA (endo and exo-glucanase) and *T. brockii* CglT (β-glucosidases). CtCelA is a thermophilic, cellulosomal enzyme belonging to GH8 family encoding a polypeptide of 488 amino acids, consisting of a signal peptide, a catalytic domain and a C-terminal dockering domain [41]. Cel6B has a similar activity to the fungal *T. reesei* exocellulase Cel6A, but with higher thermostability (optimal activity at 60 °C) and a much broader pH optimum. Its catalytic domain is linked via a linker-peptide to a carbohydrate binding module 2 (CBM2) at N-terminus, which confines the substrate and appears to modulate the activities or properties of the catalytic core [42]. CbCelA is a large multimodular enzyme complex containing N-terminal endo-β-1,4-glucanase domain belonging to GH9, three CBM3 domains and a C-terminal GH48 exo-β-1,4-glucanase domain. At elevated temperatures, CbCelA enzyme activity towards its crystalline substrate (avicel) has been reported to be seven times as high as that of the common exo- and endo-cellulase standard mixture, Cel7A and Cel5A [43, 44]. CglT encoding β-glucosidases is an intracellular protein belonging to GH1 family and studies have shown that it has a high glucose tolerance as well as thermostability [45].

Heterologous expression of the GH enzymes was under the control of the well-established promoter (P_*ldh*_) of the *G. stearothermophilus ldh* gene [23], while secretion was aided by native signal peptides identified on the cellulases used. The extracellular GHs were successfully expressed as soluble, functional proteins, including the full-length CbCelA from *C. bescii*. CbCelA has been prominent in GH research since its discovery as it effectively degrades the high as well as low-crystallinity substrates [43]. However, so far its production has only been possible in its native host and in a mesophilic heterologous host, *E. coli* [46, 47]. The production of secreted and full-length functional CbCelA in *G. thermoglucosidasius* is, therefore, one of the first time in a thermophile that heterologous production of this multi-functional enzyme has been achieved. Nevertheless, despite the employment of a strong promoter, visible detection of a protein band via Western blot required a 100-fold concentration of *G. thermoglucosidasius* culture supernatant. One possible reason for this might be that the signal peptides used were native to *C. bescii* and not optimal for secretion in the host of *G. thermoglucosidasius* [48, 49]. Alternatively, as reported in *Bacillus* species [50–52], heterologous proteins are more susceptible to proteolysis by extracellular proteases. Interestingly, the level of CbCelA protein produced was lower than that observed with CtCelA and Cel6B despite the fact that the encoded genes were expressed from the same, P_*ldh*_ promoter. It has been shown that in *C. bescii*, CbCelA is extensively glycosylated when expressed extracellularly, either as a requirement for protein translocation, or as a necessary post translational modification to shield the complex architecture of CbCelA against proteases [47]. It is probable that such a mechanism is lacking in *Geobacillus* resulting in lower levels of expression or higher level of protease cleavage once the protein is produced and secreted. Furthermore, the high prevalence of proline–threonine–serine regions found in the linkers between the CBM domains in CbCelA might also be more susceptible to proteolysis, contributing to lower expression levels [37].

Significant enzyme activities were obtained for all the heterologous expressed GH enzymes without concentrating the culture supernatant. For CtCelA and Cel6B, a specific activity of 15 and 0.0135 µmol min^−1^ mg^−1^ of protein was observed against their model substrate CMC and RAC, respectively. Finally, for CbCelA, both endo- and exo-glucanase activities were confirmed as evident by the degradation of model substrates, CMC and RAC (0.735 ± 0.07 and 0.0831 ± 0.006 μmol min^−1^ mg^−1^ of protein, respectively). It is notable that the exoglucanase activity of CbCelA was more than sevenfold higher compared to Cel6B, despite a much lower protein expression level as detected by Western blotting. This might be due to the fact that CbCelA enzyme has three CBM domains; while Cel6B has only one, which is reported to enhance the activity towards the substrates [53–55]. Several studies have reported the heterologous expression of cellulases in bacteria, fungi and yeast. However, the production of heterologous cellulases in heterologous hosts can be affected by numerous factors, including conditions of growth, heterologous secretion and the optimum temperature and pH at which the enzymes are active [56–58]. In the present study, although assays of the enzymes CtCelA, Cel6B and CbCelA were undertaken under conditions (pH 6.0 and temperature 60 °C) that were within the optimal temperature and pH ranges of these cellulases [37, 59], specific activities were low. This was most likely a consequence of using crude supernatants as opposed to purified enzymes.

For the intracellular CglT, a significant level of protein expression was observed on Western blotting despite the chromosomal location of the gene in *G. thermoglucosidasius* ZB3bInt. It is apparent from previous studies that during cellulose saccharification, cellobiose accumulation causes feedback inhibition against many exo- and endo-glucanases [60] and can often be the rate-limiting step in the hydrolytic process. Although, most of the *Geobacillus* species possess the ability to transport and metabolise disaccharides, oligosaccharides, sugar alcohols and sugar acids, *G. thermoglucosidasius* NCIMB 11955 is known to utilise cellobiose [28, 32]. However, the expression of β-glucosidase genes is invariably tightly regulated and controlled via several global regulators in most microorganisms and is often maintained in a silent state in the absence of cellobiose or other disaccharides [61–63]. As a consequence, cellobiose is utilised inefficiently. In our study, we demonstrated that the integration of CglT encoding β-glucosidase in the recombinant *G. thermoglucosidasius* ZB3Int strain led to complete degradation of 30 g/l cellobiose in 10 h; whereas, the wild-type strain failed to completely utilise the cellobiose provided.

In the present study, the exo–endo-glucanase properties of CtCelA plus Cel6B and CbCelA were combined with the β-glucosidase activity of CglT in recombinant strains BZ9 and BZ10, respectively. The growth of both strains on pre-treated wheat straw was considerably improved compared to wild type, although an initial lag phase was observed. The latter is plausible due to the imposition of a metabolic burden on the engineered strains, as a consequence of the production of the cellulose degrading enzymes. The slightly more vigorous growth of wild-type strain in the early phase of the cultures is most likely due to the utilisation of the small amount of xylose present in pre-treated wheat straw. Once this is exhausted, the cell density of wild-type cultures significantly declined. Monitoring of cellulolytic activities in BZ9 and BZ10 strains growing on pre-treated wheat straw as a sole carbon source confirmed that all the enzymes were expressed, secreted and functional.

To produce cellulosic ethanol, the biomass degrading ability of the recombinant *G. thermoglucosidasius* BZ9 and BZ10 was combined with the ethanologenic property of the *G. thermoglucosidasius* LS242 strain [26] resulting in strains BZ243 and BZ244, respectively. Production of ethanol from pre-treated wheat straw was approximately twofold and 1.6-fold higher compared to non-engineered LS242, demonstrating that at least some cellulose was converted into ethanol. Despite the fact that strains BZ243 and BZ244 were the first reported engineered *G. thermoglucosidasius* strains producing ethanol directly using cellulose obtained from industry relevant process without enzymatic treatment, the yield of ethanol is very low and far from being industrially relevant when compared with pure glucose fermentation [23]. This could be attributed to several factors. Firstly, recombinant *G. thermoglucosidasius* BZ243 and BZ244 strains were cultured for fermentation in micro-aerobic condition with no shaking and aeration, which may lead to unequal distribution of insoluble pre-treated wheat straw, compromising feedstock utilisation. Secondly, the activity of the P_ldh_ promoter is known to be threefold lower in cultures grown under micro-aerobic conditions compared to in aerobic conditions [32]. Thirdly, the effect of fermentative growth conditions on expression levels and enzyme activities, as well as the concerted action of the enzymes used, are unpredictable and hard to measure or compare to the native GHs producing organisms.

## Conclusion

In this report, we have heterologously expressed GH enzymes (CtCelA, Cel6B, CglT and CbCelA) in *G. thermoglucosidasius* both individually and synergistically with detectable activities. When grown on pre-treated wheat straw as a sole carbon source, engineered *G. thermoglucosidasius* BZ9 and BZ10 strains drastically improved growth compared to the wild-type control suggesting the combined production of these GHs facilitate lignocellulosic degradation. Further, heterologous expression of GHs in an engineered ethanologenic *G. thermoglucosidasius* LS242 strain has led to substantial increase in the production of ethanol from pre-treated wheat straw. Whilst a small step of many, this advancement is supportive of the potential of *G. thermoglucosidasius* as a cellulolytic chassis for the fermentative production of biofuel. The use of the recombinant, cellulolytic *G. thermoglucosidasius* as a process organism is not, however, limited to ethanol production but has the potential to be used to produce many other biofuels and renewable chemicals with further genetic modifications.

## Materials and methods

### Chemicals and reagents

All the chemicals and bacteriological media were purchased from Sigma-Aldrich Company Ltd (Poole, UK) or Fisher Scientific Ltd (Loughborough, UK). DNA polymerases, restriction enzymes, deoxyribonucleoside triphosphates (dNTPs), DNA markers and associated reaction buffers were sourced from Thermo Fischer Scientific (UK) or New England Biolabs Ltd. (Hitchin, UK), T4 DNA ligase from Promega (Southampton, UK). EDTA-Free Protease Inhibitor Cocktail Tablets were purchased from Roche (Mannheim, Germany).

### Bacterial strains, media and growth conditions

The bacterial strains and plasmids used in this study are listed in Table 1. *E. coli* Top10 was used as a host for plasmid constructions and routinely grown aerobically with agitation of 200 rpm at 37 °C or 30 °C in Luria-Bertani (LB) broth or agar supplemented with 50 µg/ml kanamycin or 100 µg/ml ampicillin. *G. thermoglucosidasius* strains were routinely grown on TSA agar or in 2SPYNG broth (16-g Soy peptone, 10-g Yeast extract and 5-g NaCl) supplemented with 12.5 µg/ml kanamycin. For the protein expression, recombinant *G. thermoglucosidasius* were pre-cultured overnight in 10 ml of 2SPYNG medium supplemented with 12.5 µg/ml kanamycin in 50-ml falcon constantly rotating at 250 rpm, 52 °C. Fresh pre-warmed 50-ml 2SPYNG medium in baffled conical flask (Sigma, UK) was inoculated with the overnight cultures at a starting OD600 of 0.2 and grown until late stationary phase for almost 8–9 h. For the growth experiments with different carbohydrates, engineered *G. thermoglucosidasius* strains were grown in modified ASYE [27] medium (per 1 l: 1× M9 minimal salts (sigma), 0.24-g MgSO_4_, 0.011-g CaCl_2_, 0.01-g Thiamine-HCl, 0.384-g citric acid, 27.8-mg FeSO_4_.7H_2_O, 4.6-mg NiCl_3_.6H_2_O, 3.05-mg biotin, 47.6-g HEPES, 5-g yeast extract, 1000× dilution of Trace metal mix A5 and 10-g cellobiose or pre-treated wheat straw) at 250 rpm, 55 °C. The pretreated wheat straw sample used was generated at the DBT-ICT 2G Ethanol Demonstration Facility and was kindly gifted by Institute of Chemical Technology, Mumbai. Characterization for the ethanol production on 1% pre-treated wheat straw in modified ASYE medium at 55 °C was carried out in sealed 50 ml falcon tube as previously described method [26]. For the selection of integrated genes in *G. thermoglucosidasius*, clostridial basal medium (CBM) agar supplemented with 1% w/v xylose was used [64]. All the solidified media contained 1.5% w/v agarose.

**Table 1:** List of bacterial strains and plasmids used in this study. *G. thermoglucosidasius* is abbreviated as *G*. *t*. and *E. coli* as *E. c*

Strains/plasmids	Properties	References/source
Strains		
*E. c.* Top 10	*E. coli* cloning strain	NEB
*G. t.* NCIMB 11955	wild-type strain	TMO renewables
*G. t.* 11955 *∆pyrE*	*pyrE* deletion strain of 11955	Sheng et al. [26]
*G. t.* LS004	*∆pyrE ∆ldh ∆pfl pdh* ^up^	Sheng et al. [26]
*G. t.* LS242	*∆ldh ∆pfl pdh* ^up^	Sheng et al. [26]
*G. t.* ZB1b	P_*ldh*_::Ct*celA*; Ct*celA* expressed on plasmid constitutively; Kan^R^	This study
*G. t.* ZB9a	P_*ldh*_:: *cel6B*; *cel6B* expressed on plasmid constitutively; Kan^R^	This study
*G. t.* ZB6d	P_*ldh*_::Cb*celA*; Cb*celA* expressed on plasmid constitutively; Kan^R^	This study
*G. t.* ZB3bInt	P_*ldh*_::*cglT*; *cglT* constitutively expressed in the chromosome at *pyrE* locus	This study
*G. t.* BZ9	P_*ldh*_::Cb*celA*; Cb*celA* expressed on autonomous plasmid; *cglT* constitutively expressed in the chromosome at *pyrE* locus; derived from *G. t.* ZB3bInt; Kan^R^	This study
*G. t.* BZ10	P_*ldh*_::Ct*celA*–*cel6B;* Ct*celA*–*cel6B* operon expressed on autonomous plasmid; *cglT* constitutively expressed in the chromosome at *pyrE* locus; derived from *G. t.* ZB3bInt; Kan^R^	This study
*G. t.* BZ242	P_*ldh*_::*cglT*; *cglT* constitutively expressed in the chromosome at *pyrE* locus; derived from *G. t.* LS242	This study
*G. t.* BZ243	P_*ldh*_::*CbcelA*; Cb*celA* expressed on autonomous plasmid; *cglT* constitutively expressed in the chromosome at *pyrE* locus; derived from *G. t.* BZ242 strain; Kan^R^	This study
*G. t.* BZ244	P_*ldh*_::Ct*celA*–*cel6B;* Ct*celA*–*cel6B* operon expressed on autonomous plasmid; *cglT* constitutively expressed in the chromosome at *pyrE* locus; derived from *G. t.* BZ242; Kan^R^	This study
Plasmids		
pMTLgSlimS	*G. thermoglucosidasius* shuttle vector. RepB, ColE1, Kan^R^	Sheng et al. [26]
pBSK–*ldh*	Synthesised *ldh* promoter	Biomatik
pBSK–Ct*celA*	Synthesised Ct*celA* gene	Biomatik
pBSK–*cel6B*	Synthesised *cel6B* gene	Biomatik
pBSK–*cglT*	Synthesised *cglT* gene	Biomatik
pBSK–Cb*celA*	Synthesised Cb*celA* gene	Biomatik
pMTLgSlimS–LS3	*G. thermoglucosidasius* integration vector at *pyrE* locus	Sheng et al. [26]
pJ201–Ct*celA*	Biobrick vector harbouring Ct*celA* gene flanked by *ldh* promoter	This study
pMTLgSlimS–Ct*celA*	Expression vector for Ct*celA* with P_*ldh*_ promoter	This study
pJ201–*cel6B*	Biobrick vector harbouring *cel6B* gene preceded by P_*ldh*_ promoter	This study
pMTLgSlimS–*cel6B*	Expression vector for *cel6B* with P_*ldh*_ promoter	This study
pJ201–Cb*celA*	Biobrick vector harbouring Cb*celA* gene flanked by P_*ldh*_ promoter	This study
pMTLgSlimS–Cb*celA*	Expression vector for Cb*celA* with P_*ldh*_ promoter	This study
pJ201–*cglT*	Biobrick vector harbouring *cglT* gene flanked by P_*ldh*_ promoter	This study
pMTLgSlimS–*cglT*	Expression vector for *cglT* with P_*ldh*_ promoter	This study
pMTLgSlimS–LS3–*cglT*	Integration vector for *CglT* with P_*ldh*_ promoter at *pyrE* locus	This study
pMTLgSlimS–Ct*celA*–*cel6B*	Expression vector for Ctc*elA*–*cel6B* operon with P_*ldh*_ promoter	This study

### Design and synthesis of genes encoding CtCelA, Cel6B, CglT and CbCelA

The selected GH genes; *celA* encoding endoglucanase (EC 3.2.1.4) from *Clostridium thermocellum* (designated as Ct*celA*), *cel6B* encoding exoglucanase (EC 3.2.1.91) from *Thermobifida fusca* YX, *cglT* encoding β-glucosidase (EC 3.2.1.21) from *Thermoanaerobacter brockii* and *celA* encoding multidomain cellulase (EC 3.2.1.4 and EC 3.2.1.91) from *Caldicellulosiruptor bescii* (designated as Cb*celA*) were designed as standard BioBrick-2 assembly parts. Each BioBrick-2 part consists of DNA fragment flanked by prefix (contains *Eco*RI/*Not*I/*Spe*I restriction sites) and suffix (consists of *Nhe*I/*Not*I/*Pst*I sites) sequences at 5′ and 3′ ends, respectively. In the BioBrick-2 assembly process, the combination of two parts (e.g., fusion of promoter to a gene) forms a new part which contains a scar (GCTAGT) at the junction point of the two progenitor parts while maintaining the same prefix and suffix which allows the subsequent assembly with more BioBrick-2 parts. The scar sequence represents the fusion of DNA that has been cut with *Spe*I/*Nhe*I sites and the compatible sticky ends ligated together. At the 5′-ends of each gene, BioBrick-2 prefix and the synthetic sequence 5′-TTATATTGA**AGGAGG**ATGAATGCA-3′ in which the RBS is indicated in bold was added. FLAG-epitope before the stop codon and BioBrick-2 suffix sequence was added at the 3′-ends of the Ct*celA*, *cel6B*, *cglT* and Cb*celA* gene sequences. The P_*ldh*_ promoter of the lactate dehydrogenase encoding gene (*ldh*) was synthesised without its native ribosome binding site (RBS) in the BioBrick-2 format. All the four encoding genes were codon optimised based on the genome sequence of *G. thermoglucosidasius* C56-YS93 and synthesised by Biomatik (Ontario, Canada) in the standard BB2 format. The overall configuration in synthesised BioBrick-2 format is 5′-BioBrick2prefix-syntheticRBS-*gene*-FLAG tag-BioBrick2suffix-3′. The BioBrick-2 assembled nucleotide sequences of Ct*celA*, *cel6B*, *cglT* and Cb*celA* along with the P_*ldh*_ promoter sequence used in this study are shown in Additional file 1: S1.

### Plasmid and strain constructions

The oligonucleotide primers used are listed in Additional file 1: Table S1. NEBuilder Assembly Tool was used to design primers for NEBuilder HiFi DNA Assembly reactions and purchased from Sigma-Aldrich, UK. NEBase Changer was employed to design primers specific to the mutagenesis experiment using Q5^®^ Site-Directed Mutagenesis Kit. For the amplification of the desired DNA product, either phusion High-Fidelity DNA polymerase or dream Taq Green PCR (Thermo Fisher Scientific, UK) master mix was used. PCR-amplification was performed in a standard 25–50 µl reaction using a thin walled PCR tubes placed into either a Biometra T_3000_ thermocycler (Glassgow, UK) or Sensoquest lab cycler (Geneflow, UK). Restriction enzyme digestions and PCR products were routinely separated by agarose gel electrophoresis using 0.8–1% gels prepared with analytical grade agarose (Sigma, UK) in 1xTAE buffer (40-mM Tris, 1-mM EDTA and 0.1% (v/v) glacial acetic acid). DNA fragments were purified using Zymoclean™ Gel DNA Recovery Kit (ZymoResearch, UK) following the manufacturer’s instructions. Chemically competent *E. coli* Top10 cells produced in-house were routinely used either to transform ligation reaction or complete plasmid and individual clones were selected on LB agar plates with 50 µg/ml kanamycin. Plasmids were purified using Qiagen Miniprep kit (Qiagen Ltd. Surrey, UK) according to the manufacturer’s protocol and DNA was eluted in 30–50 μl nuclease-free water (Fisher Scientific, UK) followed by storage at − 20 °C until further use. Sanger sequencing (Source BioSciences, Nottingham, UK) was routinely performed to confirm the plasmid constructs. Preparation of *G. thermoglucosidasius* electro-competent cells and electroporation procedure using Genepulser electroporator (BioRad, UK) was done in accordance with the method previously described [26]. *G. thermoglucosidasius* stock cultures were routinely maintained at − 80 °C in 12.5% (v/v) of glycerol.

#### Construction of CtcelA expression vector

All the synthesised DNA fragments were delivered in pBSK vector. The Ct*celA* gene and the P_*ldh*_ promoter synthesised in a BioBrick-2 format were assembled together in the vector pJ201 (DNA2.0) which contains the requisite prefix and suffix restriction sites for BioBrick-2 cloning. pBSK–P_*ldh*_ and pBSK–Ct*celA* were digested with *Eco*RI/*Nhe*I and *Spe*I/*Pst*I, respectively, and ligated at *Eco*RI/*Pst*I site in pJ201 vector yielding pJ201–Ct*celA* plasmid. P_*ldh*_–Ct*celA* cassette was subcloned from pJ201–Ct*celA* into pMTLgSlimS upon digestion with NotI/NheI resulting in pMTLgSlimS–Ct*celA* expression vector.

#### Construction of cel6B expression vector

pBSK–P_*ldh*_ and pBSK–*cel6B* were digested with *Eco*RI/*Nhe*I and *Spe*I/*Pst*I, respectively, and ligated at *Eco*RI/*Pst*I site in pJ201 vector yielding pJ201–*cel6B* plasmid. P_*ldh*_–*cel6B* cassette was subcloned from pJ201–*cel6B* into pMTLgSlimS upon digestion with *Not*I/*Nhe*I resulting in pMTLgSlimS–*cel6B* expression vector.

#### Construction of cglT expression vector

pBSK–P_*ldh*_ and pBSK–*cglT* were digested with *Eco*RI/*Nhe*I and *Spe*I/*Pst*I, respectively, and ligated at *Eco*RI/*Pst*I site in pJ201 vector yielding pJ201–*cglT* plasmid. P_*ldh*_–*cglT* cassette was subcloned from pJ201–*cglT* into pMTLgSlimS upon digestion with *Not*I/*Nhe*I resulting in pMTLgSlimS–*cglT* expression vector.

#### Construction of CbcelA expression vector

pBSK–P_*ldh*_ and pBSK–Cb*celA* were digested with *Eco*RI/*Nhe*I and *Spe*I/*Pst*I, respectively, and ligated at *Eco*RI/*Pst*I site in pJ201 vector yielding pJ201–Cb*celA* plasmid. P_*ldh*_–Cb*celA* cassette was subcloned from pJ201–Cb*celA* into pMTLgSlimS upon digestion with *Not*I/*Nhe*I resulting in pMTLgSlimS–Cb*celA* expression vector.

#### Construction of CtcelA–cel6B operon

For the construction of Ct*celA* and *cel6B* operon under the transcriptional control of P_*ldh*_ promoter, NEBuilder-HiFi DNA Assembly method was used according to the manufacturer’s instructions. Briefly, pMTLgSlimS plasmid was linearized by PCR amplification employing pMTLgSLimS_HiFi_F and pMTLgSLimS_HiFi_R primers (detailed sequence in Additional file 1: Table S1). The *cel6B* nucleotide sequence was amplified from plasmid pMTLgSlimS–*cel6B* using Cel6B_F and Cel6B_R pair of primers with the incorporation of new RBS-5 (TTTTAA**AGGAGG**TATAAGCT) upstream of the start codon. P_*ldh*_–Ct*celA* expression cassette was amplified from pMTLgSlimS–Ct*celA* plasmid without FLAG-tag at the C-terminal using P_ldh__CelA_F and P_ldh__CelA_R primers. All these modifications were done to avoid repetitions of FLAG-tag or same synthetic RBS in an operon and insertion of P_*ldh*_–Ct*celA*–*cel6B* cassette in *lacz* region of pMTLgSlimS vector between *Xba*I and *Hin*dIII restriction recognition sites. The PCR gel-purified pMTLgSlimS, *cel6B* and P_*ldh*_–Ct*celA* were assembled together using master mix–HiFi assembly kit according to the manufacturer’s protocol yielding pMTLgSlimS–Ct*celA*–*cel6B* expression plasmid.

### Chromosomal integration of *cglT* gene via Allele-Coupled Exchange (ACE)

The *cglT* gene along with the P_*ldh*_ promoter was subcloned from pMTLgSlims–*cglT* between the SpeI and NotI restriction sites of the integrative vector pMTLgSlimS–LS3 [26] giving rise to pMTLgSlims–LS3–*cglT.* For the integration of *cglT* gene into the chromosome at the *pyrE* locus using ACE [26], *G. thermoglucosidasius* Δ*pyrE* cells were electroporated with 1 µg of the pMTLgSLims–LS3–*cglT* plasmid DNA. Briefly, following the transformation procedure [23], cells were plated onto TSA plates supplemented with 12.5 μg/ml kanamycin and incubated overnight at 52 °C. Six large clones were then streaked on to CBM1X (CBM with 1% w/v xylose) plates lacking uracil and incubated at 62 °C for overnight. Further, single colonies were re-streaked twice on CBM1X plates without uracil to purify and incubated for 16–18 h at 62 °C. Single colonies were used as a template for colony-PCR to confirm the integration of *cglT* expression cassette with pyrE_C1_F and pyrE_C2_R primers (Additional file 1: Table S1).

### SDS-PAGE and Western blotting

The supernatants from *G. thermoglucosidasius* strains expressing extracellular recombinant CtCelA, Cel6B and CbCelA proteins were collected by centrifuging 10-ml culture at 7000 rpm for 20 min at 4 °C. Then, 1 ml of Trichloroacetic acid (TCA, 10% w/v) was added for precipitation of proteins and the suspension was stored at − 20 °C for overnight. The next day, samples were centrifuged at 10,000 rpm for 20 min at 4 °C and pellet was resuspended in 1 ml of 100 mM Tris–HCl followed by centrifugation at 13,000 rpm for 5 min at 4 °C. The washing step of pellet with 100 mM Tris–HCl was repeated three times. After the final wash, protein pellet was air dried for 60 min at room temperature and 2X NuPAGE^®^ Lithium dodecyl suphate (LDS) Sample (Thermo Fisher, UK) buffer was added to the pellet.

Cell lysate was prepared by spinning (7000 rpm for 20 min at 4 °C) 50 ml of *G. thermoglucosidasius* cells expressing recombinant CglT protein grown in 2SPYNG medium. The cell pellet was washed with 5 ml of 50-mM Tris–HCl (pH 7.8) and resuspended in 1 ml of same buffer containing EDTA-free complete protease inhibitor cocktail tablets. Cells placed in ice bath were disrupted by sonication using Soniprep 150 Ultrasonic Disintegrator (MSE Scientific Instruments, Wolflabs, UK) and the lysed cells were pelleted at 25,000×*g* for 30 min at 4 °C. The supernatant that corresponds to the soluble fraction of the cell extract was further used for SDS-PAGE or enzyme activity analysis.

All the protein samples were boiled at 95 °C for 5 min and separated by SDS-PAGE using precast 4–12% NuPAGE^®^ Bis–Tris (Thermo Fischer, UK) polyacrylamide gels according to the manufacturer’s instructions. To determine the size of the recombinant polypeptide, broad range precision plus protein™ Kaleidoscop™ pre-stained protein (Biorad, UK) marker was also loaded onto the SDS-PAGE gels. After the gel electrophoresis, proteins were electro transferred from SDS-PAGE gels onto the Trans-Blot^®^ Turbo™ Mini PVDF membranes (Bio-Rad, UK) for the western blotting. The transfer was carried out using the Trans-Blot^®^ Turbo™ Transfer System (Bio-Rad) at an electric current of 1.3 A, voltage of 25 V for 7 min. After the run was complete, membrane was blocked with 1.5% dried skimmed milk powder (Sigma, UK) in 30 ml Tris-Buffered Saline (TBS) (0.05-M Tris pH 8.0, 0.15-M NaCl) for 1 h at room temperature. The membrane was further incubated with fresh blocking solution, 1.5% dried skimmed milk powder in TBS containing at a dilution of 1:3000 monoclonal Anti-FLAG^®^ M2-peroxidase (HRP) antibody (Sigma, UK) and incubated overnight in a shaker at 4 °C. The monoclonal Anti-FLAG M2-Peroxidase is a mouse IgG antibody covalently conjugated to horseradish peroxidase (HRP) and binds to FLAG fusion proteins. After overnight incubation, membrane was washed six times with TBS containing 0.1% Tween 20 for 10 min each before developing the blot using the colorimetric 3,3′,5,5′-tetramethylbenzidine (TMB) as a substrate (Sigma, UK). After 5 min of exposure with TMB, the blot was dried and acquired for the image using scanner.

### Determination of protein concentration and enzyme activity assays

Total protein concentrations in the supernatant or soluble fraction of cell lysates were determined using BCA-kit (Thermo Fisher Scientific, UK) following the manufacturer’s instructions and standard curve generated from BSA (200–1000 µg/ml) was used to calculate the protein concentrations. For Congo-red plate staining assay, wild type and recombinant *G. thermoglucosidasius* strains producing CtCelA were streaked on TSA agar plates containing 0.5% CMC followed by incubation at 52 °C overnight. Next day, plates were flooded with 0.1% Congo red solution for 30 min at room temperature and then washed with 1-M NaCl solution [65]. RAC as a substrate for exoglucanase activity was prepared from Avicel^®^ PH-101 (Sigma, UK) as described by the method [66]. The quantitative enzyme activities of heterologous endoglucanase (CtCelA, CbCelA) and exoglucanase (Cel6B, CbCelA) were assayed with CMC (carboxy-methyl cellulose) and RAC, respectively, using the DNSA (3, 5-Dinitrosalicylic acid) method [67, 68]. Briefly, the reaction mixture containing 0.125 ml of culture supernatant (as an enzyme solution) and 0.125 ml of 1% CMC or RAC in 100 mM citrate phosphate buffer (pH 6.0) was incubated at 60 °C. Reactions were terminated after 1 and 16 h for CMC and RAC assays, respectively, by adding 0.25 ml of DNSA reagent and heating the mixture at 100 °C for 10 min. 0.1 ml of mixture was transferred into 96-well plate and the absorbance measured at 540 nm using CLARIOstar plate reader (BMG Labtech, UK). As a control, enzyme blank and substrate-independent reactions were performed simultaneously, and the absorbance determined was subtracted from the sample absorbance. One unit of enzyme activity was defined as the amount of enzyme catalysing the release of 1 µmol of reducing sugar per min from substrate under the specified assay conditions.

Synthetic chromogenic substrates *p*-nitrophenyl-β-d-glucopyranoside (pNPG) (Sigma, UK) and *p*-nitrophenyl-β-d-cellobioside (pNPC) (Sigma, UK) were used for detection of β-glucosidase (CglT) and cellobiohydrolase activity, respectively [67]. The reaction mixture containing 50-mM citrate phosphate buffer (pH 6.0), 5-mM pNPG or pNPC and 0.125-ml supernatant (used as enzyme) in a total volume of 0.55 ml was incubated at 60 °C for 10 min. After incubation 1 ml of 1% Na_2_CO_3_ was added in the mixtures to stop the reaction and absorbance measured at 400 nm with CLARIOstar plate reader (BMG Labtech, UK). Enzyme activity was calculated using calibration curve prepared with *p*-nitrophenol standard and one unit of enzyme activity is defined as the release of 1 μmol of pNP per minute under the above conditions.

### Analytical techniques

Bacterial cell growth was measured at an optical density of 600 nm (OD600) using a Novaspec II spectrophotometer (Pharmacia LKB Ltd., Cambridge, UK). Fermentation samples at various time points were collected, centrifuged at 14,000×*g* for 10 min and cell-free supernatant was used for analysis of ethanol by HPLC. Internal standard solution (80-mM valeric acid in 0.005-M H_2_SO_4_) was mixed with equal volume (0.2 ml) cell-free supernatant and filtered through 0.22-µm HPLC certified syringe filter (Whatman^®^ Spartan^®^, GE Healthcare Life Sciences, UK) and transferred into a HPLC vial with a 100 µl insert. Ethanol concentrations were quantified by Dionex UltiMate 3000 HPLC system equipped with a 300 mm × 7.8 mm Aminex^®^ HPX-87H (Bio-Rad, UK) column set at 35 °C using 5-mM H_2_SO_4_ as the mobile phase and monitored using a refractive index (RI) and Diode Array UV–Vis-detector. All the experiments were performed in triplicates and the statistically significant differences between the test samples were calculated in the PRISM (GraphPad Software, La Jolla, USA) software using Student’s *t* test or ANOVA. *P* values ≤ 0.05 were considered statistically significant.

## Additional file

**Additional file 1.** Figures S1–S5, Tables S1, File S1.

## Data Availability

All data and material used in the current study are available from the corresponding author on reasonable request.

## Abbreviations

ACE: Allele Coupled Exchange
CAZymes: Carbohydrate Active Enzymes
CBH I: cellobiohydrolase I
CBH II: cellobiohydrolase II
CBMs: carbohydrate binding module
CBM: Clostridium basal medium
CBP: consolidated bioprocessing
CCR: carbon catabolite repression
CFU: colony forming unit
CMC: carboxy-methylcellulose
DNSA: 3, 5-dinitrosalicylic acid
dNTPs: deoxyribonucleoside triphosphates
GHs: glycoside hydrolases
HRP: horseradish peroxidase
LB: Luria–Bertania medium
LDS: lithium dodecyl sulphate
OD600: optical density at 600 nm
PCR: polymerase chain reaction
pNPC: *p*-nitrophenyl-β-d-cellobioside
pNPG: *p*-nitrophenyl-β-d-glucopyranoside
RAC: regenerated phosphoric acid-swollen cellulose
RBS: ribosome binding sites
rpm: revolutions per minute
SEM: standard error mean
TBS: Tris-Buffered Saline
TCA: trichloroacetic acid
TMB: 3,3′,5,5′-tetramethylbenzidine
TSA: Tryptic Soy Agar
