# Supplementary material for: Engineering Geobacillus thermoglucosidasius for direct utilisation of holocellulose from wheat straw
Source: Biotechnol Biofuels. 2019 Aug 20;12:199. doi: 10.1186/s13068-019-1540-6 (PMC6701081; doi:10.1186/s13068-019-1540-6)
Supplement: Supplementary file 1 — Additional file 1. Figures S1–S5, Tables S1, File S1. [file 13068_2019_1540_MOESM1_ESM.docx]

**ADDITIONAL INFORMATION**

**Engineering *Geobacillus thermoglucosidasius* for direct utilization of holocellulose from wheat straw**

Zeenat Bashir^1^, Lili Sheng^1^, Annamma Anil^2^, Arvind Lali^2^, Nigel P. Minton^1^, Ying Zhang^1^*,

*^1^BBSRC/EPSRC Synthetic Biology Research Centre (SBRC), School of Life Sciences, University of Nottingham, University Park, Nottingham NG7 2RD, UK.*

*^2^DBT-ICT Centre for Energy Biosciences, Institute of Chemical Technology, Nathalal Parikh Marg, Mumbai-400019, India.*

*corresponding author: Ying.Zhang@nottingham.ac.uk

**Figure S1**

**
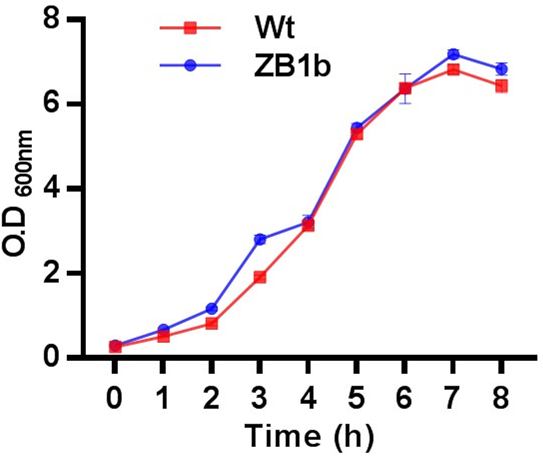
**

**Figure S1.** Growth curve analysis of wild type (WT) and recombinant *G. thermoglucosidasius* harbouring plasmid pMTLgSlimS-*CtcelA* (ZB1b). The strains were grown in 2SPYNG media at 52 ºC for 8 hours to analyse the effect of GH (CtCelA) on the growth of engineered strain.

**Figure S2**

**
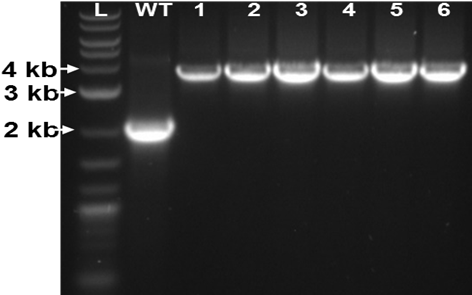
**

**Figure S2.** Integration of *cglT* gene at the *pyrE* locus of *G. thermoglucosidasius*. Gel depicting PCR screening of amplified product showing a band size of 2.0 kb for WT representing the *pyrE* gene and 3.8 kb (lane 1-6) for recombinant *G*. *thermoglucosidasius* ZB3bInt strains having the P*_ldh_*-*cglT* cassette integrated; L is DNA ladder (0.1 – 10 kb).

**Figure S3**

**
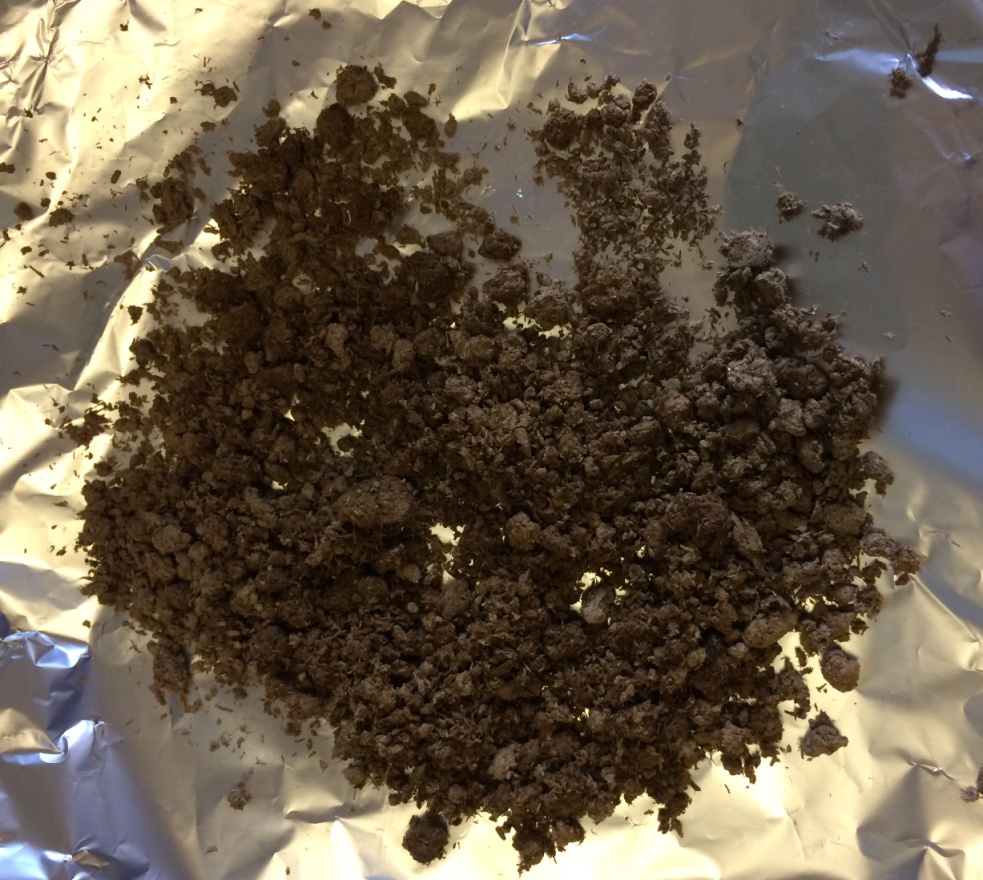
**

**Figure S3. The picture of pretreated wheat straw**. After a two-step process using nitric acid followed by ammonia, the pretreated wheat straw is composed of 85.15% cellulose and 4.35% xylose, of the total carbohydrates as reported in the patent (Lali, 2016). The biomass was sampled from the Cellulosic Alcohol Technology Demonstration Plant at India Glycols Ltd., Kashipur, India, Jan 2017.

**Figure S4**

**
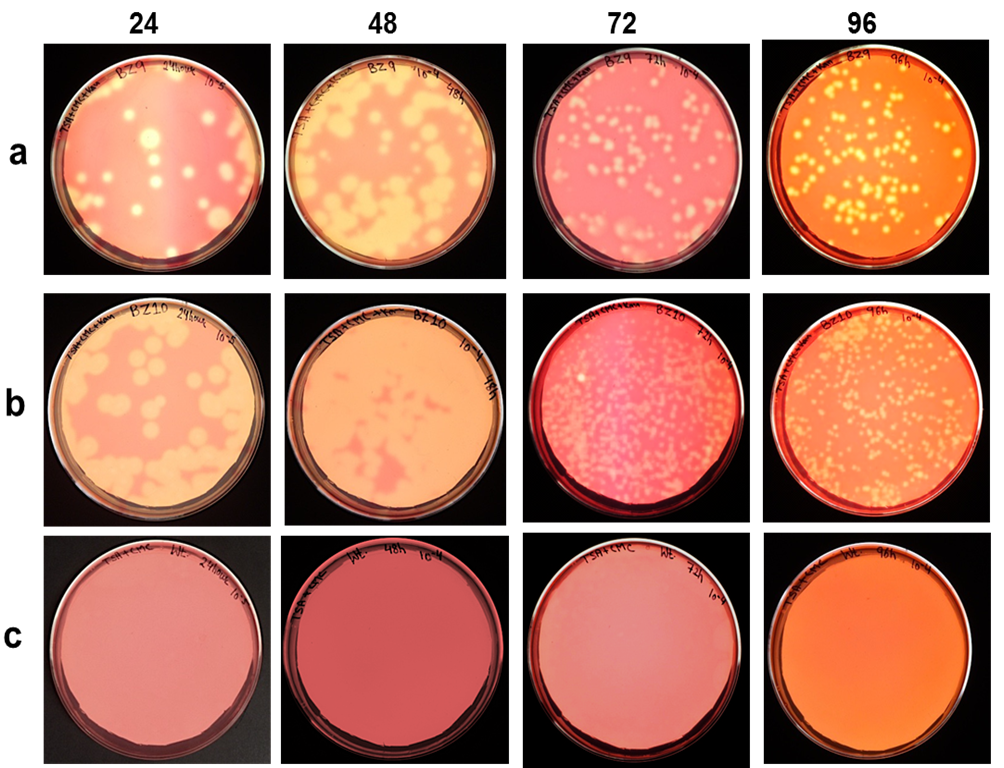
**

**Figure S4.** Congo red staining of recombinant *G. thermoglucosidasius* strains grown on pretreated wheat straw for 24-96 hours and plated on TSA agar supplemented with CMC for enzyme activity. (a) Recombinant *G. thermoglucosidasius* BZ9 strain expressing CglT and *Cb*CelA (b) *G. thermoglucosidasius* BZ10 strain expressing CglT, *Ct*CelA and Cel6B (c) *G. thermoglucosidasius* wild type strain.

**Figure S5**

**
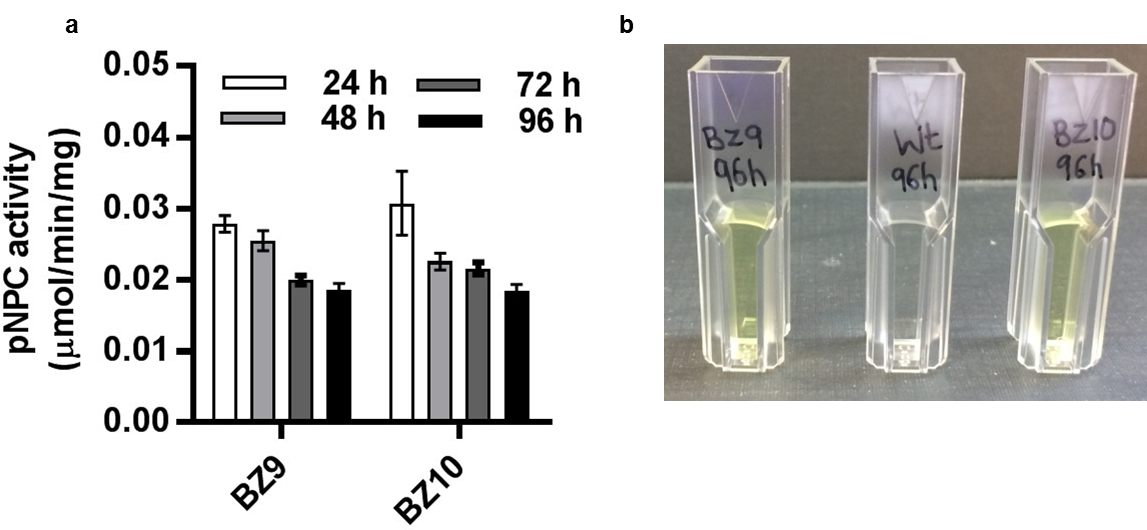
**

**Figure S5.** Colorimetric pNPC enzyme assay during growth of recombinant *G. thermoglucosidasius* BZ9 and BZ10 strains on pretreated wheat straw. (a) pNPCase specific activity of extracellular fraction of recombinant *G. thermoglucosidasius* BZ9 and BZ10 strains at 24, 48, 72, and 96 hours using pNPC (b) photograph showing pNP, a yellow colour end product liberated due to the hydrolysis of pNPC at 96 hours by *G. thermoglucosidasius* BZ9, *G. thermoglucosidasius* BZ10 and wild-type strain (Wt). Results are shown as mean ± SEM of three biological replicates.

**Table S1**

The list of primers used in this study.

| **Name Sequence (5`-3`)** |
| --- |

| pyrE_C1_F | CCCATGCTGAAAATCCAGCTG |
| --- | --- |
| pyrE_C2_R | CGGGTCGACAGAATTGTTCG |
| Cel6B_F | CCCGTATTAATTTTAAAGGAGGTATAAGCTATGAGTAAAGTTCGTGCC |
| Cel6B_R | CGACGGCCAGTGCCAAGCTTTTATTTGTCATCGTCATCTTTATAATC |
| P_ldh__CelA_F | GTACCCGGGGATCCTCTAGAGCGGGACGGGGAGCTGAG |
| P_ldh__CelA_R | GCTTATACCTCCTTTAAAATTAATACGGGAGGTGTGGAATGGATTTAATAAGATACCG |
| pMTLgSLimS_HiFi_F | TAAAAGCTTGGCACTGGCCGT |
| pMTLgSLimS_HiFi_R | TCTAGAGGATCCCCGGGTAC |

**File S1**

Colour coding in the nucleotide sequences denotes different parts of Bio-Brick assembled together.

Yellow; Nucleotide sequence of modified P*_ldh_* promoter

Red; SCAR sequence

Turquoise; Synthetic RBS

Grey; gene sequence

Pink; FLAG-epitope

P*_ldh_*-*CtcelA*

GCGGCCGCACTAGTGCGGGACGGGGAGCTGAGTGCTCCCGTTGTTTGCCGCGGCGTCTGTCATGAAATGGACAAACAATAGTCAAACAATCGCCACAATCGCGCATGCATTGCGGTGCGCCTTTCGCGTAAAATATTTATATGAAAGTGTTCGCAGCTAGTTTATATTGAAGGAGGATGAATGCAATGAAAAACAGGGTAATTTCATTATTAATGGCTTCCTTGCTTTTGGTTTTGTCGGTAATTGTTGCTCCTTTTTACAAAGCGGAAGCCGCGGGAGTGCCGTTTAATACGAAATATCCGTATGGACCTACGTCCATTGCGGATAATCAATCGGAAGTAACAGCGATGTTGAAAGCGGAATGGGAAGATTGGAAAAGCAAACGCATTACGAGCAATGGCGCGGGAGGATACAAACGGGTTCAACGTGATGCTAGTACAAATTATGATACAGTGTCCGAAGGCATGGGCTATGGCTTGCTTCTTGCGGTCTGCTTTAACGAACAAGCGTTGTTTGATGATCTTTATCGCTATGTTAAAAGCCATTTTAATGGCAATGGCCTTATGCATTGGCATATTGATGCGAATAACAATGTCACATCCCATGATGGGGGGGATGGAGCGGCGACAGATGCGGATGAAGATATTGCGTTAGCGCTTATTTTTGCGGATAAACTTTGGGGCAGCTCCGGCGCGATTAATTATGGACAAGAAGCGCGCACACTTATTAACAACTTGTATAATCATTGTGTAGAACATGGCTCGTATGTCTTGAAACCGGGCGATCGCTGGGGAGGCAGCTCGGTCACGAATCCTTCGTATTTTGCGCCAGCGTGGTATAAGGTCTATGCGCAATATACGGGGGATACACGCTGGAATCAAGTCGCGGATAAATGCTATCAAATTGTAGAAGAAGTTAAAAAATATAACAACGGCACAGGCTTAGTACCTGATTGGTGCACAGCGTCCGGCACGCCTGCGTCCGGCCAAAGCTATGATTACAAGTATGATGCGACACGGTATGGCTGGCGTACAGCGGTAGATTATAGCTGGTTTGGAGATCAACGTGCGAAAGCGAATTGTGATATGTTGACAAAATTCTTTGCGAGAGATGGAGCGAAAGGCATTGTCGATGGCTATACGATTCAAGGCTCCAAAATTTCCAACAACCATAATGCGTCGTTTATTGGACCAGTCGCGGCGGCGAGCATGACGGGCTATGATTTGAATTTTGCGAAAGAACTTTATAGAGAAACAGTCGCGGTCAAAGATTCGGAATATTATGGCTATTATGGAAATTCCCTTCGGTTACTTACGTTGTTGTATATTACGGGCAATTTTCCTAATCCACTTTCCGATCTTTCGGGCCAACCGACGCCACCTAGCAATCCTACACCTAGCCTTCCACCTCAAGTAGTCTATGGAGATGTCAATGGCGATGGCAATGTGAATAGCACGGATCTTACAATGTTGAAACGCTATCTTCTTAAATCGGTGACGAATATTAATCGTGAAGCGGCGGATGTGAATAGAGATGGCGCGATTAATTCCTCCGATATGACGATTCTTAAACGGTATCTTATTAAATCCATTCCACACCTCCCGTATGATTATAAAGATGACGATGACAAATAA

P*_ldh_*-*cel6B*

GCGGCCGCACTAGTGCGGGACGGGGAGCTGAGTGCTCCCGTTGTTTGCCGCGGCGTCTGTCATGAAATGGACAAACAATAGTCAAACAATCGCCACAATCGCGCATGCATTGCGGTGCGCCTTTCGCGTAAAATATTTATATGAAAGTGTTCGCAGCTAGTTTATATTGAAGGAGGATGAATGCAATGAGTAAAGTTCGTGCCACGAACAGACGTTCGTGGATGCGGCGCGGCCTGGCAGCCGCCTCTGGACTGGCGCTTGGCGCCTCCATGGTGGCGTTCGCTGCTCCGGCCAACGCCGCCGGCTGCTCGGTGGACTACACGGTCAACTCCTGGGGTACCGGGTTCACCGCCAACGTCACCATCACCAACCTCGGCAGTGCGATCAACGGCTGGACCCTGGAGTGGGACTTCCCCGGCAACCAGCAGGTGACCAACCTGTGGAACGGGACCTACACCCAGTCCGGGCAGCACGTGTCGGTCAGCAACGCCCCGTACAACGCCTCCATCCCGGCCAACGGAACGGTTGAGTTCGGGTTCAACGGCTCCTACTCGGGCAGCAACGACATCCCCTCCTCCTTCAAGCTGAACGGGGTTACCTGCGACGGCTCGGACGACCCCGACCCCGAGCCCAGCCCCTCCCCCAGCCCTTCCCCCAGCCCCACAGACCCGGATGAGCCGGGCGGCCCGACCAACCCGCCCACCAACCCCGGCGAGAAGGTCGACAACCCGTTCGAGGGCGCCAAGCTGTACGTGAACCCGGTCTGGTCGGCCAAGGCCGCCGCTGAGCCGGGCGGTTCCGCGGTCGCCAACGAGTCCACCGCTGTCTGGCTGGACCGTATCGGCGCCATCGAGGGCAACGACAGCCCGACCACCGGCTCCATGGGTCTGCGCGACCACCTGGAGGAGGCCGTCCGCCAGTCCGGTGGCGACCCGCTGACCATCCAGGTCGTCATCTACAACCTGCCCGGCCGCGACTGCGCCGCGCTGGCCTCCAACGGTGAGCTGGGTCCCGATGAACTCGACCGCTACAAGAGCGAGTACATCGACCCGATCGCCGACATCATGTGGGACTTCGCAGACTACGAGAACCTGCGGATCGTCGCCATCATCGAGATCGACTCCCTGCCCAACCTCGTCACCAACGTGGGCGGGAACGGCGGCACCGAGCTCTGCGCCTACATGAAGCAGAACGGCGGCTACGTCAACGGTGTCGGCTACGCCCTCCGCAAGCTGGGCGAGATCCCGAACGTCTACAACTACATCGACGCCGCCCACCACGGCTGGATCGGCTGGGACTCCAACTTCGGCCCCTCGGTGGACATCTTCTACGAGGCCGCCAACGCCTCCGGCTCCACCGTGGACTACGTGCACGGCTTCATCTCCAACACGGCCAACTACTCGGCCACTGTGGAGCCGTACCTGGACGTCAACGGCACCGTTAACGGCCAGCTCATCCGCCAGTCCAAGTGGGTTGACTGGAACCAGTACGTCGACGAGCTCTCCTTCGTCCAGGACCTGCGTCAGGCCCTGATCGCCAAGGGCTTCCGGTCCGACATCGGTATGCTCATCGACACCTCCCGCAACGGCTGGGGTGGCCCGAACCGTCCGACCGGACCGAGCTCCTCCACCGACCTCAACACCTACGTTGACGAGAGCCGTATCGACCGCCGTATCCACCCCGGTAACTGGTGCAACCAGGCCGGTGCGGGCCTCGGCGAGCGGCCCACGGTCAACCCGGCTCCCGGTGTTGACGCCTACGTCTGGGTGAAGCCCCCGGGTGAGTCCGACGGCGCCAGCGAGGAGATCCCGAACGACGAGGGCAAGGGCTTCGACCGCATGTGCGACCCGACCTACCAGGGCAACGCCCGCAACGGCAACAACCCCTCGGGTGCGCTGCCCAACGCCCCCATCTCCGGCCACTGGTTCTCTGCCCAGTTCCGCGAGCTGCTGGCCAACGCCTACCCGCCTCTGGATTATAAAGATGACGATGACAAATAA

P*_ldh_*-*CbcelA*

GCGGCCGCACTAGTGCGGGACGGGGAGCTGAGTGCTCCCGTTGTTTGCCGCGGCGTCTGTCATGAAATGGACAAACAATAGTCAAACAATCGCCACAATCGCGCATGCATTGCGGTGCGCCTTTCGCGTAAAATATTTATATGAAAGTGTTCGCAGCTAGTTTATATTGAAGGAGGATGAATGCAATGAAACGCTATCGTCGCATTATTGCGATGGTAGTGACGTTTATTTTTATTCTTGGAGTTGTCTATGGAGTGAAACCGTGGCAAGAAGTTCGTGCGGGCTCGTTTAATTATGGAGAAGCGTTGCAAAAAGCGATTATGTTTTATGAATTTCAAATGTCCGGCAAATTACCGAATTGGGTTCGTAATAACTGGCGGGGGGATTCCGCGCTTAAAGATGGACAAGATAACGGCCTTGATCTTACTGGGGGCTGGTTTGATGCGGGCGATCATGTGAAGTTCAATCTTCCGATGTCGTATACAGGAACGATGCTTAGCTGGGCGGTGTATGAATATAAGGATGCGTTTGTGAAATCGGGACAATTAGAACATATTCTTAATCAAATTGAATGGGTCAATGATTATTTTGTGAAATGTCATCCATCCAAATATGTCTATTATTATCAAGTTGGAGATGGCAGCAAAGATCATGCGTGGTGGGGACCAGCGGAAGTCATGCAGATGGAACGCCCGTCGTTTAAAGTCACACAATCGAGCCCAGGCAGCACGGTAGTTGCGGAAACAGCGGCGTCCCTTGCGGCGGCGTCCATTGTCCTTAAAGATCGTAATCCTACGAAAGCGGCGACATATCTTCAACATGCGAAAGAACTTTATGAATTTGCGGAAGTCACAAAATCGGATGCGGGCTATACAGCGGCGAATGGCTATTATAATAGCTGGTCCGGCTTTTATGATGAACTTAGCTGGGCGGCGGTCTGGTTGTATCTTGCGACGAATGATAGCACATATCTTACGAAAGCGGAATCGTATGTTCAAAATTGGCCTAAAATTTCCGGCTCCAATACGATTGATTATAAGTGGGCGCATTGCTGGGATGATGTGCATAACGGTGCGGCGTTGTTGCTTGCGAAAATTACAGGCAAAGATATATATAAGCAAATTATTGAATCCCATCTTGATTATTGGACAACAGGCTATAACGGTGAACGCATTAAATATACGCCTAAAGGACTTGCGTGGTTAGATCAATGGGGCTCGTTACGGTATGCGACAACAACGGCGTTTCTTGCGTTTGTGTATTCGGATTGGGTAGGCTGCCCTTCCACTAAAAAGGAAATATATCGCAAATTTGGAGAATCCCAAATTGATTATGCGCTTGGCTCCGCGGGCCGCTCCTTTGTAGTAGGCTTTGGCACGAATCCACCTAAACGCCCACATCATCGCACAGCGCATAGCTCCTGGGCGGATAGCCAATCCATTCCTTCGTATCATAGACATACGTTGTATGGCGCGCTTGTCGGCGGACCAGGCTCGGATGATAGCTATACAGATGATATTAGCAATTATGTTAATAACGAAGTCGCGTGTGATTATAATGCGGGCTTTGTGGGTGCGCTTGCGAAAATGTATCAACTTTATGGAGGCAATCCTATTCCTGATTTTAAAGCGATTGAAACACCTACGAATGATGAGTTTTTCGTAGAAGCGGGCATTAATGCGTCCGGCACGAATTTTATTGAAATTAAAGCGATTGTTAACAATCAATCGGGCTGGCCTGCGCGTGCGACGGATAAACTTAAATTTCGCTATTTTGTAGACCTTTCGGAACTTATTAAAGCGGGCTATAGCCCGAATCAACTTACGTTGAGCACGAATTATAATCAAGGCGCGAAAGTCTCGGGACCTTATGTGTGGGATGCGTCCAAAAATATATATTATATTCTTGTAGATTTTACAGGCACACTTATATACCCCGGTGGACAAGATAAATATAAGAAAGAAGTTCAATTTCGCATTGCGGCGCCTCAAAATGTGCAATGGGATAATAGCAATGATTATAGCTTTCAAGATATTAAAGGTGTAAGCTCCGGATCGGTAGTGAAAACGAAATATATTCCGTTGTATGATGGCGATGTCAAAGTTTGGGGAGAAGAACCTGGCACGTCCGGCGCGACACCGACACCAACGGCGACGGCGACGCCTACACCAACGCCTACGGTCACACCTACACCTACACCTACGCCTACGAGCACAGCGACACCGACGCCAACACCTACGCCAACAGTAACACCTACACCAACGCCGACGCCTACAGCGACACCTACAGCGACACCAACACCGACGAGCACGCCTTCCTCCACACCAGTCGCGGGAGGCCAAATTAAAGTGTTGTATGCGAACAAAGAAACGAATAGCACAACGAATACGATTCGCCCGTGGCTTAAAGTAGTCAATACAGGATCGTCTAGCATTGATCTTTCCCGCGTAACGATTCGCTATTGGTATACAGTCGATGGAGATAAAGCGCAATCCGCGATTTCCGATTGGGCGCAAATTGGCGCGTCCAATGTCACGTTTAAATTTGTTAAACTTAGCAGCTCGGTATCCGGAGCGGATTATTATCTTGAAATTGGCTTTAAATCGGGCGCGGGACAACTTCAAGCGGGCAAAGATACGGGAGAAATTCAAATTCGCTTTAACAAGTCCGATTGGTCCAATTATAATCAAGGCAATGATTGGTCCTGGATGCAATCCATGACGAATTATGGAGAAAATGTCAAAGTAACAGCGTATATTGATGGAGTACTTGTCTGGGGACAAGAACCGTCCGGCGCGACACCAACACCTACGGCGACGCCTGCGCCTACAGTAACACCTACACCTACGCCTACACCAACGTCCACGCCTACAGCGACGCCTACGGCGACGCCAACACCTACGCCGACGCCGTCGAGCACACCAGTCGCGGGAGGACAAATTAAAGTACTTTATGCGAATAAGGAAACGAATAGCACAACGAATACGATTAGACCGTGGTTAAAAGTAGTTAATACAGGATCAAGCAGCATTGATCTTAGCAGAGTAACGATTCGCTATTGGTATACAGTAGATGGAGATAAAGCGCAATCCGCGATTTCCGATTGGGCGCAAATTGGAGCGTCGAATGTCACGTTTAAATTTGTCAAACTTTCATCGTCGGTCTCCGGAGCGGATTATTATCTTGAAATTGGCTTTAAATCGGGCGCGGGACAACTTCAAGCGGGCAAAGATACGGGAGAAATTCAAATTCGCTTTAATAAGTCGGATTGGTCGAATTATAATCAAGGCAATGATTGGTCGTGGATGCAATCCATGACGAATTATGGAGAAAATGTTAAAGTCACGGCGTATATTGATGGAGTGTTAGTCTGGGGCCAAGAACCGTCCGGCGCGACACCAACACCGACAGCGACACCTGCGCCTACAGTAACGCCGACGCCGACGCCTGCGCCTACACCGACGCCAACACCGACGCCGACAGCGACGCCAACACCAACGCCGACGCCGACGCCGACGGCGACACCTACGGTGACAGCGACGCCGACACCAACACCGTCGAGCACGCCTTCGGTGCTGGGGGAATATGGCCAACGGTTTATGTGGTTGTGGAATAAGATTCATGATCCTGCGAATGGCTATTTTAATCAAGATGGCATTCCGTATCATTCGGTAGAAACGTTGATTTGTGAAGCGCCAGATTATGGACATTTGACAACATCGGAAGCGTTTTCCTATTATGTCTGGTTAGAAGCGGTGTATGGCAAACTTACCGGAGATTGGTCCAAATTCAAAACAGCGTGGGATACGTTAGAAAAATATATGATTCCTTCCGCGGAAGATCAACCGATGAGATCGTATGATCCGAACAAGCCTGCGACATACGCGGGAGAATGGGAAACACCAGATAAATATCCATCCCCGTTAGAGTTCAATGTGCCAGTGGGCAAAGATCCTCTTCATAACGAGTTAGTCTCCACATACGGCTCCACGTTGATGTATGGTATGCATTGGTTGATGGATGTCGATAATTGGTATGGCTATGGCAAACGGGGAGATGGAGTCTCCCGCGCGTCCTTTATTAATACGTTTCAACGGGGCCCTGAAGAATCGGTGTGGGAAACCGTGCCTCATCCATCGTGGGAAGAGTTCAAATGGGGAGGCCCTAATGGCTTTCTTGATCTTTTTATTAAAGATCAAAATTATAGCAAACAATGGCGCTATACAGATGCGCCAGATGCGGATGCGAGAGCGATTCAAGCGACGTATTGGGCGAAAGTCTGGGCGAAAGAACAAGGCAAATTCAATGAGATTTCGAGCTATGTAGCGAAAGCGGCGAAAATGGGAGATTATCTTCGCTATGCGATGTTTGATAAATATTTTAAACCGTTAGGCTGCCAAGATAAAAATGCGGCGGGCGGCACAGGCTATGATTCCGCGCATTATCTTCTTAGCTGGTATTATGCGTGGGGTGGTGCGTTAGATGGCGCGTGGTCGTGGAAAATTGGCAGCTCCCATGTGCATTTTGGCTATCAAAATCCTATGGCGGCGTGGGCGCTTGCGAATGATTCGGATATGAAACCTAAAAGCCCTAATGGTGCGTCGGATTGGGCGAAAAGCCTTAAACGTCAAATTGAATTTTATCGCTGGTTACAATCCGCGGAAGGTGCGATTGCGGGGGGAGCGACGAATAGCTGGAATGGCCGCTATGAAAAATATCCTGCGGGAACAGCGACGTTTTATGGCATGGCGTATGAACCTAATCCAGTCTATCATGATCCAGGCTCCAATACATGGTTTGGCTTTCAAGCGTGGTCCATGCAACGGGTCGCGGAATATTATTATGTCACTGGAGATAAAGATGCGGGAGCGTTGTTAGAAAAATGGGTGAGCTGGGTGAAATCCGTAGTGAAACTTAATTCGGATGGCACGTTTGCGATTCCGTCCACACTTGATTGGTCCGGCCAACCTGACACCTGGAATGGCGCGTATACAGGCAATAGCAATCTTCATGTCAAAGTAGTCGATTATGGCACGGATCTTGGCATTACGGCGTCCTTAGCGAATGCGCTTTTGTATTATTCCGCGGGCACGAAAAAATATGGAGTCTTTGATGAAGGCGCGAAAAATCTAGCGAAAGAACTTCTTGATCGCATGTGGAAACTTTATAGAGATGAAAAAGGCCTTTCCGCGCCAGAAAAACGCGCGGATTATAAGCGGTTCTTTGAACAAGAAGTCTATATTCCTGCGGGCTGGATTGGCAAAATGCCTAACGGGGACGTTATTAAATCCGGAGTGAAATTCATTGATATTCGCAGCAAATACAAGCAAGATCCTGATTGGCCTAAACTTGAAGCGGCGTACAAGTCCGGCCAAGCGCCAGAATTTCGCTATCATCGCTTTTGGGCGCAATGTGATATTGCGATTGCGAATGCGACCTATGAAATTTTATTTGGCAATCAAGATTATAAAGATGACGATGACAAATAA

P*_ldh_*-*cglT*

GCGGCCGCACTAGTGCGGGACGGGGAGCTGAGTGCTCCCGTTGTTTGCCGCGGCGTCTGTCATGAAATGGACAAACAATAGTCAAACAATCGCCACAATCGCGCATGCATTGCGGTGCGCCTTTCGCGTAAAATATTTATATGAAAGTGTTCGCAGCTAGTTTATATTGAAGGAGGATGAATGCAATGATTATTGAAATTGGAGTAATTAAACTTGCGAAATTTCCTAGAGATTTTGTCTGGGGCACAGCGACTAGCTCCTATCAAATTGAGGGGGCGGTCAACGAGGATGGACGTACGCCGTCCATTTGGGATACGTTTAGCAAAACGGAAGGCAAAACGTATAAGGGACATACCGGAGATGTCGCGTGTGATCATTATCATCGCTACAAAGAAGATGTCGAAATTCTTAAAGAAATTGGAGTCAAAGCGTATCGCTTTAGCATTGCGTGGCCTAGAATTTTTCCTGAAGAAGGCAAATATAATCCGAAAGGCATGGATTTTTATAAGAAACTTATTGATGAACTTCAAAAACGGGATATTGTACCTGCGGCGACGATATACCATTGGGATCTTCCACAATGGGCGTATGATAAAGGTGGAGGCTGGTTGAATCGGGAATCCATTAAATGGTATGTAGAATATGCGACGAAACTTTTTGAAGAACTTGGCGATGCGATTCCGTTGTGGATTACACATAACGAGCCGTGGTGCAGCTCCATTCTTAGCTATGGCATTGGAGAACATGCGCCGGGCCATAAAAATTATCGGGAAGCGCTTATTGCGGCGCATCATATTTTGTTGTCTCATGGCGAAGCGGTCAAAGCGTTTCGTGAGATGAATATTAAAGGCTCCAAAATTGGCATTACACTTAATCTTACACCAGCGTATCCTGCGTCCGAAAAAGAAGAAGATAAACTTGCGGCGCAATATGCGGATGGCTTTGCGAATCGCTGGTTTCTTGATCCGATTTTTAAAGGCAATTATCCGGAAGATATGATGGAACTTTATTCCAAAATTATTGGAGAATTTGATTTTATTAAAGAAGGGGATCTTGAAACAATTTCGGTCCCTATTGATTTTCTTGGAGTCAATTATTATACACGCTCGATTGTCAAATATGATGAAGATTCTATGCTTAAAGCGGAAAATGTACCAGGACCAGGCAAACGCACAGAAATGGGCTGGGAAATTAGCCCGGAATCGTTGTATGATCTTCTTAAACGGTTAGATAGAGAATATACGAAACTTCCTATGTATATTACGGAAAATGGCGCGGCGTTTAAAGATGAAGTAACAGAAGATGGCCGGGTACATGATGATGAACGCATTGAATATATTAAAGAACATCTTAAAGCGGCGGCGAAATTCATTGGCGAAGGTGGCAATCTTAAAGGCTATTTTGTGTGGTCGCTGATGGATAATTTTGAATGGGCGCATGGCTATAGCAAACGGTTTGGCATTGTCTATGTCGATTATACGACACAAAAACGCATTCTTAAAGATAGCGCGTTGTGGTACAAGGAAGTCATTCTTGATGATGGCATTGAAGAT GATTATAAAGATGACGATGACAAATAA
